# Reactive Capture and Conversion of Carbon Dioxide to Methanol with ZnZrO_2_ and Alkali-Promoted Mg_3_AlO_*x*_ Mixed Oxide Catalytic Sorbents

**DOI:** 10.1021/acssuschemeng.4c10562

**Published:** 2025-03-21

**Authors:** Laura Proaño, Katlo Galefete, Guanhe Rim, Gabriel Gusmão, Christopher W. Jones

**Affiliations:** School of Chemical & Biomolecular Engineering, Georgia Institute of Technology, Atlanta, Georgia 30332, United States

**Keywords:** CO_2_ capture, CO_2_ hydrogenation, reactive capture and conversion, catalytic sorbents, alkali-modified catalysts

## Abstract

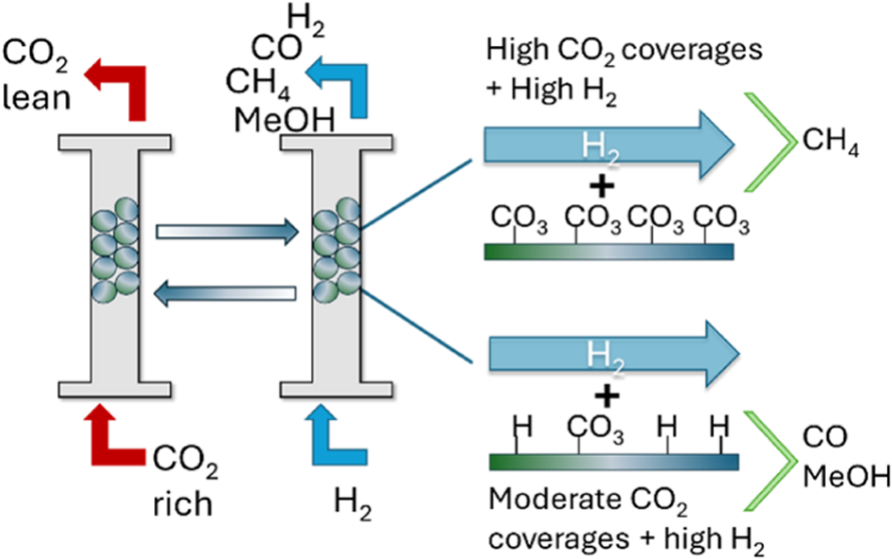

Reactive capture and conversion (RCC) explores the use of a single-unit process to capture CO_2_ and produce a product, in this case, methanol (MeOH). In this study, different configurations of a catalytic sorbent (CS) composed of ZnZrO_2_ catalyst and Mg_3_AlO_*x*_ sorbent with and without alkali modification are evaluated for CO_2_ adsorption, steady-state catalysis with cofed CO_2_ and H_2_, and transient RCC performance. A catalyst composed of a physical mixture of Mg_3_AlO_*x*_ with ZnZrO_2_ resulted in a slight increase in CO_2_ uptake, with a low impact on the catalytic activity and RCC of the materials compared to ZnZrO_2_ alone. In contrast, Na impregnation significantly increased the level of CO_2_ uptake from 0.28 mmol/g (ZnZrO_2_ alone) to 0.6 and 1.1 mmol/g for the CS with Na on the catalyst or Mg_3_AlO_*x*_, respectively. However, Na impregnation reduced the CO_2_ conversion rate and MeOH selectivity during steady-state cofeed experiments at 300 °C and 6 bar. In contrast to steady-state catalysis conditions, RCC, which is a cyclic capture and conversion process, creates dynamic CO_2_ and H_2_ surface coverages, favoring CH_4_ in the early stages of the conversion step and then CO and MeOH as the catalyst CO_2_ coverage reduces. The highest MeOH productivity during RCC was achieved with CS that balanced the CO_2_ uptake with only moderate catalyst rate reductions caused by Na addition. The optimal material, ZnZrO_2_+10%Na/Mg_3_AlOx, achieved a CO_2_ uptake of 0.8 mmol/g and a MeOH productivity of 0.5 mmol/g with 100% selectivity at 260 °C and 6 bar during RCC. This marks the highest RCC MeOH productivity reported to date, although the process needs further optimization and even with optimization, may remain impractical. The results further demonstrate that optimization of catalytic sorbents under steady-state flow conditions does not easily correlate to transient capture and conversion cycles for methanol synthesis from CO_2_.

## Abstract 2

Catalytic sorbent configurations were evaluated for reactive capture and conversion of CO_2_ to CH_3_OH.

## 1 Introduction

The continuing increase in CO_2_ atmospheric concentrations, currently at ∼420 ppm, is pushing the need for developing net-zero CO_2_ technologies that can supply the demand for C-based products.^1^ A potential approach is carbon capture and utilization, in which CO_2_ is captured from a point source or form air, and then is converted to valuable C-based products such as syngas (CO+H_2_), CH_4_, CH_3_OH, or higher hydrocarbons. One of the limitations of this approach is the energy-intensive process for desorption, purification, and compression of CO_2_.^2^ For that reason, in recent years, a process intensification approach based on reactive capture and conversion (RCC) using dual function materials or catalytic sorbents (CS) has been explored to conduct capture and conversion over the same material in a 2-step process.^3^

Various chemistries have been investigated for the RCC using catalytic sorbents. These include the reverse water gas shift reaction (RWGS) (eq 1),^4,5^ dry reforming of methane (DRM) (eq 2),^6^ methanation (eq 3),^6,7^ methanol synthesis (eq 4),^2,8^ and higher hydrocarbon production.^9^ For these processes, various catalytic sorbent configurations have been studied: (1) depositing a CO_2_-sorbing phase onto a catalyst by impregnating a metal-based catalyst with an alkali salt precursor,^2,10^ (2) physically mixing a CO_2_ adsorbent and a catalyst,^11^ and (3) employing a double-bed configuration with a CO_2_ adsorbent bed followed by a catalyst bed.^8^ Despite these advancements, the literature remains unclear on the specific benefits of various CS configurations and their influence on CO_2_ uptake, catalytic performance, and overall RCC activity and productivity
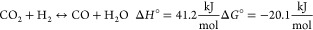
1
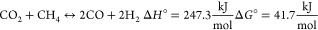
2
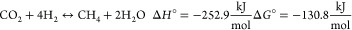
3
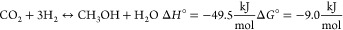
4
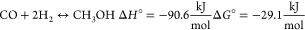
5

For the synthesis of CO via the RWGS reaction or DRM, or for CH_4_ production via CO_2_ methanation, the most common CS configuration used in the literature is the impregnation of the catalyst with an alkali or alkaline salt precursor, and it is believed that close contact between the sorbent and the catalyst sites is critical for high productivity. However, Park et al. reported a higher CH_4_ productivity during RCC with a CS composed of separate phases of Ru/Al_2_O_3_ and NaNO_3_/MgO using a two-bed configuration, as opposed to a mixed and pelletized configuration, in which the catalyst and adsorbent are in close contact. The authors hypothesized that the high loading of NaNO_3_ (∼10 wt %) might poison or block the catalytic active sites when the two materials are in close contact.^11^ Porta et al. also evidenced poisoning of the catalytic active site when conducting cofeed methanation experiments over an alkali-impregnated Ru/Al_2_O_3_ catalyst. However, during RCC experiments, these materials had high CH_4_ productivity, despite the loss in CO_2_ conversion and CH_4_ selectivity. This suggests that the CO_2_ storage capacity of the material is more critical during the RCC process than the MeOH synthesis rate.^12^

For MeOH production, Wirner et al. used a dual bed composed of Na/Al_2_O_3_ and Cu/ZnO/Al_2_O_3_ catalyst (CZA). The authors chose this approach due to prior reports of alkali poisoning of the CZA catalyst.^8,13^ The authors reported a MeOH productivity of 12 μmol/g_cs_ at 230 °C and 9 bar, with a 26% MeOH selectivity and 74% CO selectivity.^8^ In the physical mixture configuration, MeOH productivity decreased to 2 μmol/g_cs_, and the authors indicated that this could be related to the oxidation of the CZA catalyst during the CO_2_ adsorption. The dual-bed setup favors catalyst rereduction during the conversion step, as H_2_ saturates the bed before CO_2_ desorption, which would not occur in close-contact configurations. On the contrary, Potter et al. reported a higher MeOH productivity (∼50 μmol/g) using a 5 wt % alkali K-impregnated CZA catalyst. During cofeed experiments, the K-promoted CS suppressed MeOH formation and decreased CO_2_ conversion compared to the CZA catalyst. Interestingly, during RCC experiments, K-impregnated CS had the highest MeOH productivity, which aligns with this material having the highest CO_2_ uptake. Moreover, Wirner et al. and Potter et al. both reported the formation of CH_4_ during RCC across all of the materials and configurations, even though during cofeed experiments, no CH_4_ formation was observed.^2,8^

The observed shifts in product distribution when comparing RCC versus cofeed steady-state catalysis operation may stem from the distinct reaction conditions of the two cases, leading to different coverages of reactive intermediates on the catalyst surface(s). Specifically, during RCC, excess H_2_ is present compared to steady-state catalysis conditions. If the conditions over the catalyst are sufficiently different, the distribution of surface intermediates may change enough to favor different reaction pathways and thus altered product distributions. In the case of methanation, *in situ* diffuse reflectance infrared Fourier transform spectroscopy (DRIFTS) studies have shown different intermediate species over a Na–Ru/Al_2_O_3_ CS as opposed to the related Ru/Al_2_O catalyst.^14,15^ Park et al. conducted kinetic and steady-state isotopic transient kinetic analysis (SSITKA) experiments over 10%NaNO_3_/Ru/Al_2_O_3_ catalyst and observed a change of the reaction mechanism during cofeed methanation compared to the Ru/Al_2_O_3_ catalyst.^11,16^ Over the Na-impregnated CS, bidentate carbonates, formates, and linear carbonyl species were the most likely intermediates, while for the Ru/Al_2_O_3_, bicarbonate and linear carbonyl species were the most likely intermediates. However, it is not clear if this change is significant during RCC operation, since the 2-bed configuration was the most productive in that study, and in that case, the different domains are not in close contact. In the case of MeOH synthesis through RCC, preliminary *in situ* DRIFTS experiments conducted by Potter et al. also revealed different adsorbed surface species for the K-CZA CS, as opposed to the unpromoted CZA CS materials. For K-CZA, an additional bidentate carbonate signal was identified, while for CZA, only the bridge and polycarbonates were identified. Since K-CZA had the highest uptake, the authors hypothesized that its RCC activity could be related to this different binding mode of CO_2_.^2^

Finally, other authors reported the beneficial effect of mixing a CZA catalyst with Mg-based hydrotalcite (Mg_3_AlO_*x*_) during CO_2_ hydrogenation to MeOH cofeed experiments. Fang et al. reported an enhanced catalytic MeOH space-time yield (STY) for a 40 wt % hydrotalcite-CZA mixture. They ascribed the increase in catalytic activity to an increase in CO_2_ partial pressure near the CZA catalyst domain, due to CO_2_ adsorption on the Mg_3_AlO_*x*_domain.^17^ The authors did not explore RCC over these materials, though the increased CO_2_ uptake and observed catalytic activity suggest that such materials may be suitable for RCC to MeOH.

In this work, we explore the RCC of CO_2_ to MeOH using CS composed of ZnZrO_2_ as the catalytic domain and Mg_3_AlO_*x*_ hydrotalcite-derived mixed metal oxide as the CO_2_ adsorbent. The effect of alkali impregnation and the CS configuration are studied and expressed in terms of the CO_2_ adsorption capacity, cofeed steady-state CO_2_ hydrogenation rates, and RCC rates. The different configurations allowed us to understand the main factors driving the RCC rate. Finally, RCC is studied via *in situ* DRIFTS to identify possible changes in surface intermediates during RCC over the ZnZrO_2_ catalyst, ZnZrO_2_+Mg_3_AlO_*x*_ CS, and alkali-promoted CS.

## 2 Materials and Methods

### 2.1 Catalytic Sorbents Preparation

#### 2.1.1 ZnZrO_2_ Catalysts

ZnZrO_2_ catalyst (ZZO) with a Zn:Zr molar ratio of 1:6 and Mg_3_Al-CO_3_ hydrotalcite with an Mg:Al molar ratio of 3:1 were synthesized by coprecipitation following methodologies reported in the literature.^18−21^ Detailed description can be found in the Supporting Information.

#### 2.1.2 Catalytic Sorbents

Three configurations of catalytic sorbents (CS) were prepared. First, a physical mixture of ZnZrO_2_+Mg_3_AlO_*x*_ in a mass ratio of 6:4 was prepared, and the material was manually ground and mixed for 10 min. Two more CS were prepared, the first where the alkali precursor (NaNO_3_) was impregnated directly on the ZZO catalyst or on the Mg_3_Al-CO_3_ sorbent. A 1:1 acetone: water solution of 1 M NaNO_3_ was prepared, and then the corresponding amount was added to the support to achieve a 10 wt % loading of Na_2_O after calcination. Next, the material was calcined at 400 °C in static air, and finally, it was physically mixed with the corresponding nonimpregnated counterpart to obtain 10%Na/ZZO+Mg_3_AlO_*x*_ and ZZO+10%Na/Mg_3_AlO_*x*_ in a mass ratio of catalyst to sorbent of 6:4.

### 2.2 Catalyst Characterization

The properties of the materials were characterized by using various techniques: Brunauer–Emmett–Teller (BET) surface area measurements, X-ray powder diffraction (XRD), and temperature-programmed methods (CO_2_-TPD and H_2_-TPR). Detailed experimental procedures and additional characterization information are provided in the Supporting Information.

*In situ* FT-IR spectroscopy was performed on a Nicolet iS10 IR spectrometer with a high-temperature DRIFTS cell (Harrick Scientific Products, Inc.). After sample reduction in H_2_ at 400 °C, the temperature was ramped to 300 °C. Then, CO_2_ adsorption was conducted using 10% CO_2_/N_2_ and desorption was conducted in a H_2_ atmosphere. Spectra were collected with 64 scans and with a resolution of 4 cm^–1^.

### 2.3 CO_2_ Adsorption Experiments

CO_2_ uptake was recorded in a Netzsch TGA instrument (STA449F3 Jupiter). Approximately 50 mg of the sample was placed on an alumina sample pan. All samples were *in situ* activated at 400 °C for 2 h under N_2_ (90 mL/min) and then the temperature was cooled to 300 °C and equilibrated for 30 min. The feed gas was switched to 10%CO_2_/N_2_ (90 mL/min) for 90 min. The mass change was recorded dynamically, and the CO_2_ uptake was calculated from the mass increase. Additionally, breakthrough experiments were performed in a stainless-steel fixed-bed tubular reactor. For this, samples were prereduced in H_2_ at 400 °C for 2 h and cooled to 300 °C in N_2_, after which the feed was switched to 10% CO_2_/N_2_ for 1 h and the outlet of the reactor was recorded in a Quantek IR gas analyzer (Model 906). The breakthrough capacity was calculated from the integrated area between the CO_2_ profile in a SiC-filled reactor and the CO_2_ profile obtained from the different CS (eq S1). Following breakthrough experiments, reactive capture and conversion (RCC) experiments were conducted, as explained in section 2.5.

### 2.4 Steady-State Catalytic Activity

The catalytic activity of the different catalytic sorbent configurations at steady state under different temperatures, total pressure, and weight hourly space velocity (WHSV) was evaluated in a 1/4″ 316 stainless-steel fix-bed reactor tube contained within 12″ homemade aluminum blocks that are heated by two 550 W Chromalox cartridge heaters. A cylinder with 10% CO_2_, 33% H_2_, and balance N_2_ ± 2% (Mathenson) was used as the feed. The reactor inlet flow rate was controlled using a mass flow controller (MFC) (Brooks Instruments) and the pressure was controlled by a back-pressure regulator (TESCOM, 26-1700) coupled with an electronic regulator (ER3000). The outlet stream was analyzed by an online 7890 Agilent gas chromatograph (GC) equipped with two TCD and one FID detector. An additional set of experiments was carried out to estimate the apparent H_2_ and CO_2_ reaction order. In these experiments, the H_2_ or CO_2_ partial pressure was varied while maintaining the other reactant partial pressure fixed, and the flow of N_2_ was adjusted to maintain a WHSV of 7200 mL/min at 300 °C and 6 bar.

For all experiments, 0.3–0.5 g of the pelletized and sieved (425–125 μm) catalytic sorbent were placed between 2 beds of SiC and were *in situ* reduced under H_2_ at 400 °C for 2 h. After reduction, the reactor was cooled to the temperature of interest and pressurized to the pressure of interest using the targeted CO_2_/H_2_/N_2_ mixture. For all of the experiments, data were collected every 15 min and steady state was reached when the integrated areas of the peaks in the GC chromatogram did not change by more than 1%. The carbon balance was always 100 ± 4%.

### 2.5 Reactive Capture and Conversion

Reactive capture and conversion (RCC) experiments were carried out in the same setup used for the steady-state catalytic activity experiments. Usually, 0.5 g of material was loaded into the reactor and *in situ* reduced in H_2_ 400 °C for 2 h, then the reactor was cooled to the desired reaction temperature. The feed was switched to 10% CO_2_/N_2_ and held for 1 h. After CO_2_ capture, the feed was switched to N_2_ and the reactor was purged for 5–7 min. Then, the feed was switched to pure H_2_, and the reactor was pressurized to the pressure of the interest. For all steps, CO_2_ capture, purge, and H_2_ conversion, a total flow of 38 mL/min was used. Once the reactor reached the set pressure, the effluent was analyzed using the GC instrument, and the conversion step was run until no more products were detected. The RCC productivity and product distribution were estimated from the area under the curve for each product flow rate estimated from the GC.

## 3 Results and Discussion

### 3.1 Characterization of the Catalytic Sorbents

**Figure 1:**
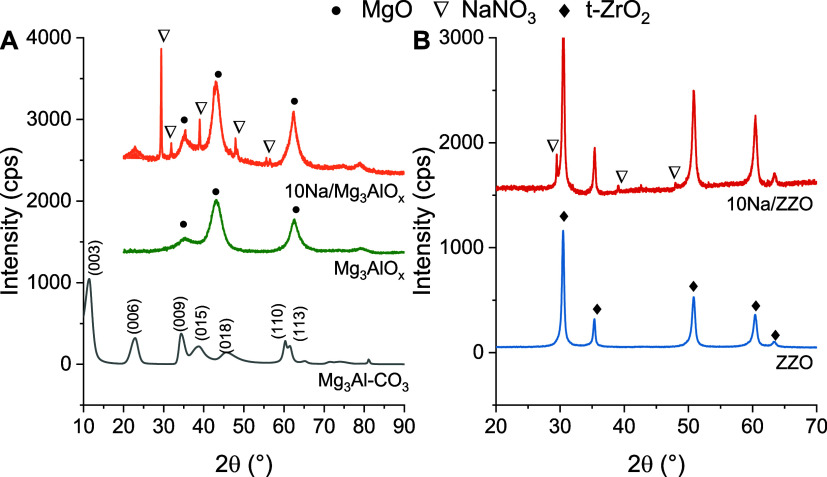
(A) XRD patterns of fresh Mg_3_Al-CO_3_ hydrotalcite, calcined hydrotalcite, and calcined, 10%NaNO_3_ impregnated hydrotalcite. (B) XRD patterns of the ZnZrO_2_ catalyst and 10%NaNO_3_ impregnated catalyst.

Figure 1A presents the XRD patterns of the as-synthesized Mg_3_Al-CO_3_ hydrotalcite, post-calcination Mg_3_AlO_*x*_ mixed metal oxide, and Na-impregnated Mg_3_AlO_*x*_ sorbents. For the as-synthesized Mg_3_Al-CO_3_, characteristic peaks of the layered double hydroxide appeared with lamellar basal peaks (003, 006, and 009) at 11.3 and 22.8° and nonbasal peaks (015, 018, 110, and 113) at 34.1, 38.5, 45.8, 60.2, and 61.6°.^22,23^ After calcination at 400 °C, the LDH peaks disappeared, and broad peaks at 35.3, 43.1, and 62.6° corresponding to crystal planes (111), (200), and (220), respectively, of MgO appeared, indicating the collapse of the layered structure. For the 10% NaNO_3_/Mg_3_AlO_*x*_ sorbent sample, characteristic peaks of NaNO_3_ appeared at 29.3, 31.8, 39.9, 47.8, and 48.4 °. Figure 1B presents XRD patterns on the ZZO catalyst and 10% NaNO_3_/ZnZrO_2_. In the case of the ZZO catalyst, only peaks related to t-ZrO_2_ appeared and no ZnO was detected, suggesting that ZnO is in small-size clusters or inserted in the ZrO_2_ lattice forming a solid solution.^19^

The N_2_ isotherms of the Mg_3_AlOx adsorbents, ZZO, and CS are presented in Figure S2, with the textural properties in Table 1. For the fresh Mg_3_AlOx, calcined, and impregnated samples, the adsorption isotherms were type II according to IUPAC classification for macroporous materials. Moreover, the Mg_3_Al-CO_3_ samples showed no adsorption limitations at high relative pressures and a H3 hysteresis loop was identified, which is usually found in materials with plate-like particles and slit-shaped pores, consistent with hydrotalcite materials.^24−26^ Fresh Mg_3_Al-CO_3_ hydrotalcite samples had a BET surface area of 111 m^2^/g, which increased to 219 m^2^/g after calcination. This increase in BET surface area is related to the structural collapse after dehydration and decarbonation that occurs during calcination at 400 °C (Figure S1). However, the increase in the BET surface area is lost after impregnation with 10%NaNO_3_, with the surface area decreased to 57 m^2^/g. The ZZO catalyst (Figure 1B) presents a type IV N_2_ isotherm typical of mesoporous materials, with a low BET surface area of 44 m^2^/g, which is consistent with literature reports.^19^ In the CS samples, the N_2_ isotherm is dominated by the Mg_3_AlO_*x*_ sorbent, resulting in a Type II isotherm. This suggests that ZnZrO_2_ could be distributed in small aggregates within the Mg_3_Al-CO_3_ sorbent; this was also verified by STEM-EDS (Figure S3).

**Table 1:** Textural Properties of Fresh and Calcined Mg_3_AlO_*x*_ Adsorbents, ZnZrO_2_ Catalyst, and Catalytic Sorbents

sample	BET surface area (m^2^/g)	BJH pore volume (cm^3^/g)
Mg_3_Al-CO_3_	113	0.53
Mg_3_AlO_*x*_	219	0.88
10% NaNO_3_/Mg_3_AlO_*x*_	57	0.26
ZnZrO_2_	44	0.05
ZnZrO_2_+Mg_3_AlO_*x*_	66	0.20
10%NaNO_3_/ZnZrO_2_+Mg_3_AlO_*x*_	31	0.12
ZnZrO_2_+10%NaNO_3_/Mg_3_AlO_*x*_	35	0.29

**Figure 2:**
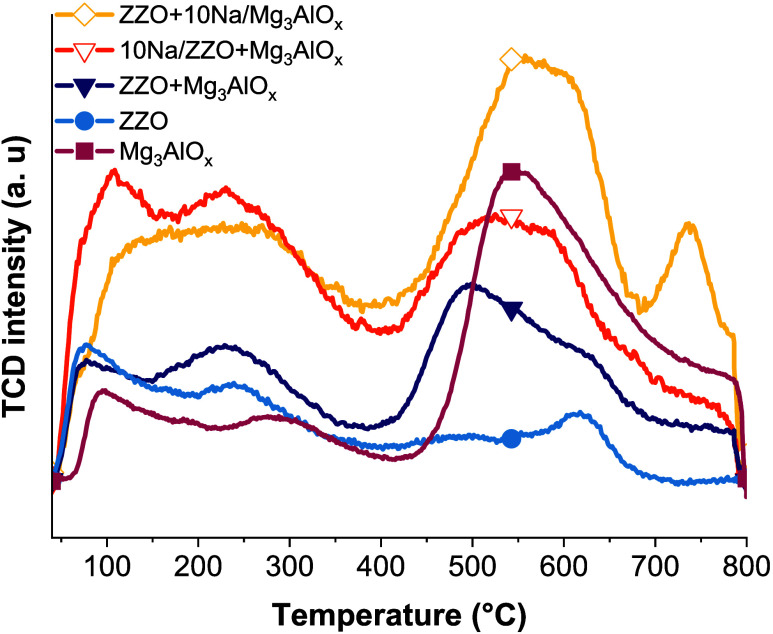
CO_2_-TPD profiles of the different sorbents, catalysts, and the 10%NaNO_3_-impregnated CS.

The basic strength of the materials was evaluated by CO_2_ TPD, and the desorption profiles are presented in Figure 2. Several desorption peaks between 100 and 800 °C appeared, with desorption peaks between 100 and 300 °C assigned to weakly and moderately bound CO_2_ and peaks between 400 and 500 °C to strongly bound CO_2_. The ZZO catalyst has mostly weak-moderate strength CO_2_ adsorption sites, to which its high CO_2_ hydrogenation activity has been related.^19,27,28^ On the contrary, the Mg_3_AlOx material presented mostly strong adsorption sites, with a desorption peak around 550 °C. The ZZO+Mg_3_AlO_*x*_ presented the contributions of both ZnZrO_2_ and Mg_3_AlOx. Finally, for the NaNO_3_-impregnated samples, an increase in all basic sites was observed.

### 3.2 CO_2_ Adsorption over the CS

**Figure 3:**
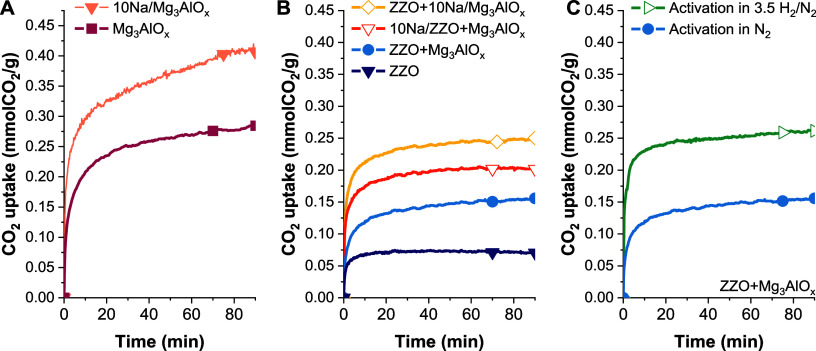
TGA CO_2_ uptakes for (A) Mg_3_AlO_*x*_ sorbent and 10%NaNO_3_ promoted sorbent and (B) the ZnZrO_2_ catalyst, as well as CS composites, under 10%CO_2_/N_2_ at 300 °C, and (C) ZnZrO_2_+Mg_3_AlO_*x*_ activated in N_2_ and 3.5% H_2_/N_2_.

The CO_2_ adsorption performance of the Mg_3_AlO_*x*_ sorbent, ZZO catalysts, and 10%NaNO_3_ CS composites was evaluated by TGA under 10% CO_2_/N_2_. After the N_2_ pretreatment, Mg_3_AlO_*x*_ had a CO_2_ uptake of 0.27 mmol/g, which agrees with literature reports for mixed metal oxides (MMO) derived from hydrotalcites with an Mg/Al ratio of 3 at 300 °C.^24^ Impregnation of the Mg_3_AlO_*x*_ with 10 wt % NaNO_3_ resulted in an increased CO_2_ adsorption capacity of 0.42 mmol/g; this enhancement is related to the increased basicity of the material, as is widely reported in the literature.^24,29,30^Figure 3B presents the CO_2_ uptake of the ZZO catalyst and the CS composites. ZZO has a minimal CO_2_ uptake, 0.07 mmol/g, after pretreatment in N_2_. Incorporation of 40 wt % of the adsorbent phase into the catalyst results in an increased CO_2_ adsorption capacity to 0.15, 0.20, and 0.25 mmol CO_2_/g, for ZZO+Mg_3_AlO_*x*_, 10Na/ZZO+Mg_3_AlO_*x*_, and ZZO+10Na/Mg_3_AlO_*x*_, respectively. An increase in the CO_2_ uptake from 0.16 to 0.20 mmolCO_2_/g when pretreatment is done under 3.5% H_2_/N_2_ atmosphere was observed for the ZZO+Mg_3_AlO_*x*_ (Figure 3C) material. This increase could be related to the reducibility of ZZO, as shown in H_2_-TPR (Figure S4). During reduction, Zn can be inserted into the ZrO_2_ lattice, which creates oxygen vacancies active for CO_2_ adsorption.^19,31^ Thus, the total uptake of CS when the sample is activated in H_2_ at 400 °C was estimated from fixed-bed breakthrough experiments (Table 2), rather than via TGA.

**Table 2:** CO_2_ Uptake of Different CS Estimated by TGA, Fix-Bed, and CO_2_-TPD

	**CO**_**2**_**uptake (mmolCO**_**2**_**/g**)	
	**TGA**	**fix-bedbreakthrough**^a^
ZZO	0.05	0.25
Mg_3_AlO_*x*_	0.27	0.27
ZZO+Mg_3_AlO_*x*_	0.15	0.30
ZZO+10Na/Mg_3_AlO_*x*_	0.25	1.13
10Na/ZZO+Mg_3_AlO_*x*_	0.20	0.48

a After reduction at 400 °C in H_2_

### 3.3 Catalytic Activity during CO_2_+H_2_ Cofeed Experiments

**Figure 4:**
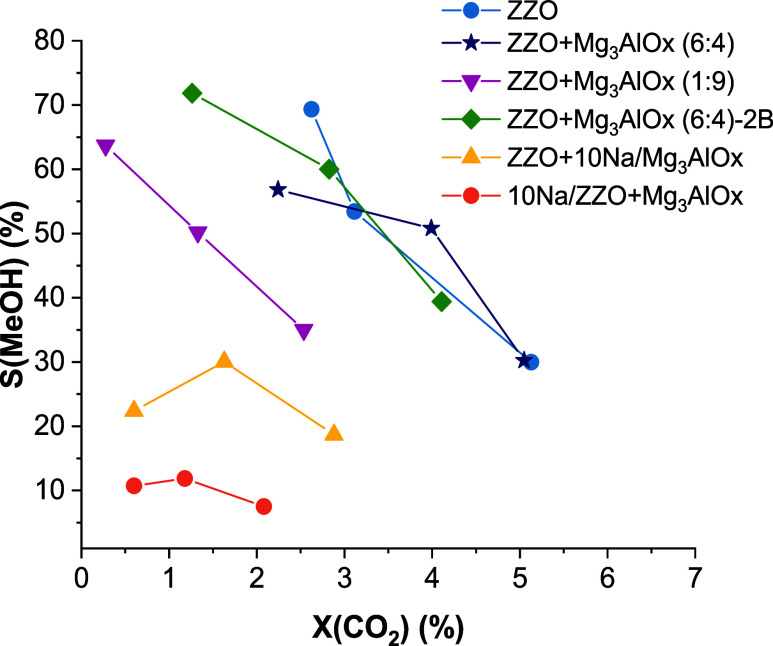
Conversion vs selectivity for CO_2_ hydrogenation to MeOH for different ZnZrO_2_+Mg_3_AlO_*x*_ CS configurations during steady-state cofeed experiments. Reaction conditions: 300 °C, 10 bar, and WHSV between 2500 and 16200 mL/g_cat_/h.

The CS composites were evaluated under steady-state CO_2_ hydrogenation conditions, i.e., cofeed experiments with a H_2_:CO_2_ ratio of 3:1, to assess the impact of the CS configuration. Figure 4 presents the CO_2_ conversion vs MeOH selectivity at 300 °C and 6 bar. For the low-Mg_3_AlO_*x*_-content material, 40 wt % Mg_3_AlO_*x*_, the presence of the Mg_3_AlO_*x*_ did not modify the catalytic activity, but for the high-Mg_3_AlO_*x*_-content sample, ZZO:Mg_3_AlO_*x*_ of 1:9, the MeOH selectivity decreased from 70 to 30% at a CO_2_ conversion of about 3% compared to the ZZO catalyst alone. Fang et al. reported a similar behavior for the MeOH yield when adding a commercial hydrotalcite to a CZA catalyst, with an optimum content of 40 wt %.^17^ For the CS impregnated with 10%NaNO_3_, a decrease in the CO_2_ conversion and MeOH selectivity was observed at similar WHSVs from 2500 to 16,200 mL/g_cat_/h. The decrease in CO_2_ conversion could be related to catalyst poisoning, blocking of the active site(s), or promotion of other reaction pathways that lead to CO formation. This negative effect on the CO_2_ conversion and MeOH selectivity was more significant when the NaNO_3_ was impregnated directly on the ZZO catalyst, as opposed to when it was impregnated on the Mg_3_AlO_*x*_.

To further understand the effect of Na impregnation, apparent reaction order experiments were conducted at 300 °C and 6 bar. Then, nonlinear regression to a power law rate eq (eq Supporting Information) was carried out using fitnml in Matlab. The estimated apparent reaction orders are presented in Table 3 and Figure S5. For the MeOH synthesis reaction and RWGS, the CO_2_ apparent reaction order was close to 0 for the ZZO catalyst, which agrees with previous reports of Cu-based catalysts.^28,32,33^ For MeOH synthesis, the H_2_ apparent reaction order increased from 1.5 for the ZZO catalyst to 1.6 and 2.0 for the Na-impregnated CS, indicating that MeOH formation is highly sensitive to the availability of H_2_. On the other hand, the formation of CO by RWGS has a lower dependency on H_2_ partial pressure. Thus, the decrease in MeOH selectivity over the Na-impregnated CS catalysts can result from poisoning of H_2_ activation sites, which is more significant when Na is impregnated directly to the catalyst vs the sorbent phase.

**Table 3:** Apparent Reaction Order for ZnZrO_2_ and 10% NaNO_3_ Promoted CS from Steady-State Cofeed Experiments

	**apparent reaction order**			
	MeOH		RWGS	
**sample**	CO_2_	H_2_	CO_2_	H_2_
ZnZrO_2_	0.0	1.5	0.1	0.6
ZnZrO_2_+10%NaNO_3_/Mg_3_AlO_*x*_	0.0	1.6	0.2	0.4
10%NaNO_3_/ZnZrO_2_ + Mg_3_AlO_*x*_	0.2	2.0	0.4	0.1

The negative impact of alkali promotion on MeOH selectivity was attributed to a change in the relevant reaction intermediates on the surface. For example, Bansode et al. evaluated the effect of 5 wt % K and Ba impregnation on a Cu/Al_2_O_3_ catalyst during CO_2_ hydrogenation experiments at 200 °C, 100 bar, H_2_:CO_2_ ratio of 3.8:1 and GHSV of 4000 h^–1^.^13^ They reported a MeOH selectivity of 45% for the Cu/Al_2_O_3_ catalyst, which decreased to 5% when the catalyst was impregnated with K, while Ba promoted the MeOH selectivity to 65%. Through *in situ* DRIFTS experiments, the authors evidenced the formation of bidentate carbonates over the K-promoted catalyst, while for the Ba-promoted Cu/Al_2_O_3_ catalyst, mainly bicarbonate and formate species were identified. Thus, the authors suggested that K poisons the Cu active site surface and favors the formation of RWGS reaction intermediates. Potter et al. also observed similar trends over K- and Ca-promoted CZA catalysts. They reported that K shifted the MeOH selectivity, while Ca did not have any effect on it. They attributed this to the different CO_2_ binding modes identified by *in situ* DRIFTS, in which the CZA catalyst and Ca/CZA exhibited the same bands related to bridged bidentate carbonates and polydentate carbonates, while the K/CZA catalyst had only bidentate carbonate bands. While the differences observed in the *in situ* DRIFTS spectra between the unpromoted catalyst and alkali-impregnated catalysts were associated with the observed changes in CO_2_ conversion and MeOH selectivity, it is also possible that the visible adsorbed species are not all active intermediates and some may be spectators outside the catalytic cycle. Thus, the current literature still lacks a comprehensive understanding of the change in product distribution during RCC with alkali-impregnated CS. Further operando and kinetic experiments are needed to assess the reactivity of the adsorbed species and their role as intermediates in reaction pathways.

### 3.4 In Situ DRIFTS Evaluation

To evaluate the adsorbed modes of CO_2_ over the catalytic sorbents and the evolution of surface carbon species under a H_2_ atmosphere, *in situ* DRIFTS experiments using ZZO, 10Na/Mg_3_AlO_*x*_, and ZZO+10Na/Mg_3_AlO_*x*_ mimicking RCC conditions were conducted. For ZZO, a broad band appears during the CO_2_ capture step from 1100 to 1900 cm^–1^. Upon deconvolution using a Gauss function, 7 peaks can be identified at 1071, 1228, 1355, 1442, 1507, 1584, and 1688 cm^–1^. The peaks at 1355 and 1507 cm^–1^ and at 1688 and 1355 cm^–1^ were assigned to O–C–O *v*_3_ vibrations of monodentate and bidentate carbonates (CO_3_*), respectively.^19,27,34^ The peaks at 2966, 2870, 1584, and 1442 cm^–1^ were assigned to formate (HCOO*) species.^27^ The peaks at 2820 and 2930 cm^–1^ were assigned to methoxy (CH_3_O*) species.^27,32^ It has been reported in the literature through operando *in situ* DRIFTS, DFT, and kinetic studies that MeOH synthesis follows the HCOO* mediated pathway (e.g., HCOO* → HCOOH* → H_2_COOH* → H_2_CO* → CH_3_O*) during steady-state cofeed operation for ZZO and Cu/CeOx/TiO_2_ catalysts.^19,32,35,36^Figure S6 shows the time evolution of the band intensities during the CO_2_ capture and conversion step. During the CO_2_ capture step, the CO_3_* and HCOO* band intensities increase and become stable; when the feed is switched to H_2_ for the conversion step, the CO_3_* band intensities rapidly decrease, while the HCOO* bands increase and then slowly decrease. Additionally, during the conversion step, CH_3_O* bands slowly appear. Thus, it can be speculated that during RCC, CO_3_* species are hydrogenated to form more HCOO*, which then transforms to CH_3_O*. However, further operando or isotopic studies are required to conclusively elucidate if CO_3_* species are indeed important reaction intermediates (or spectators).

**Figure 5:**
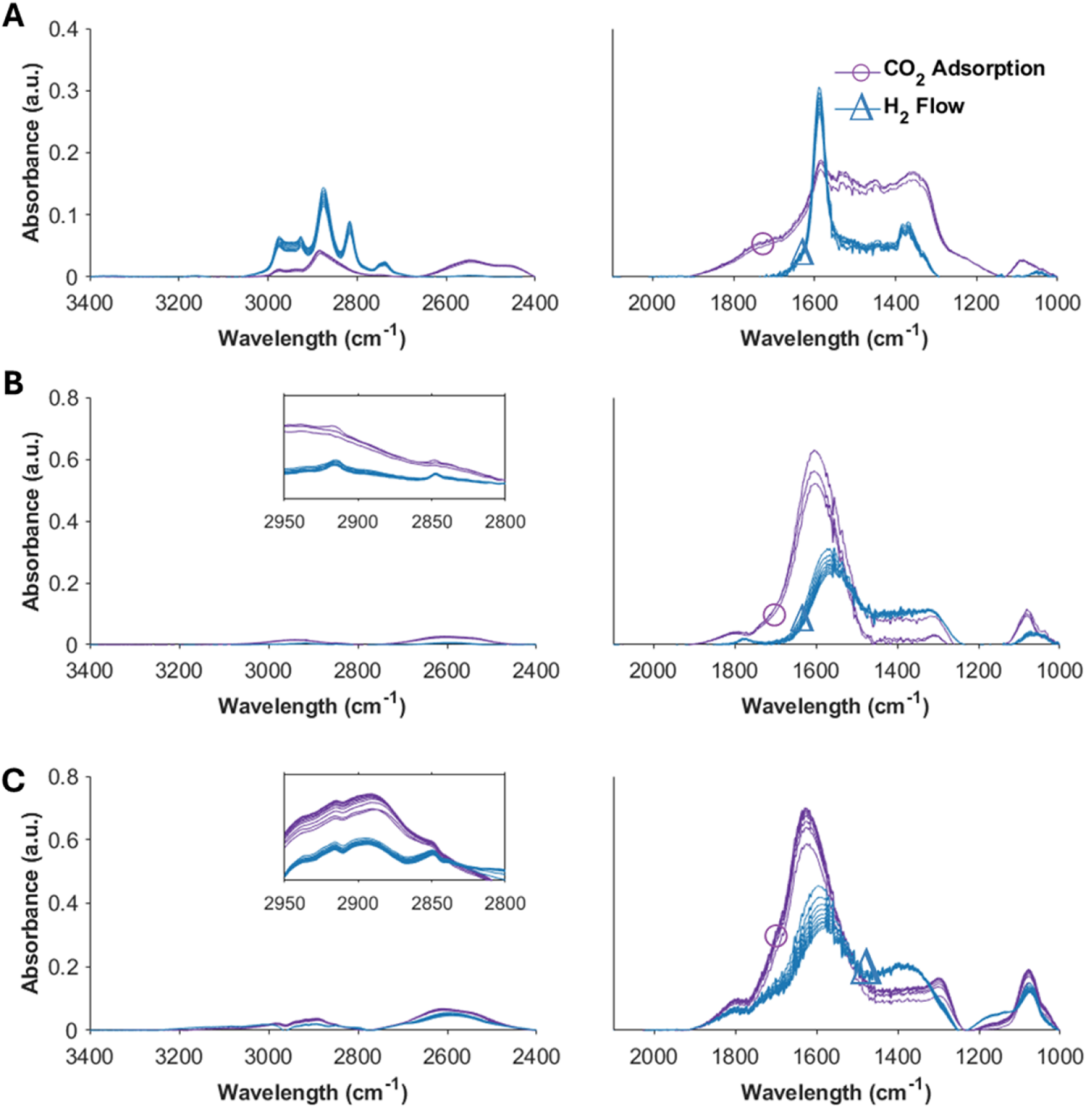
*In situ* DRIFTS spectra during CO_2_ capture and conversion over the (A) ZZO catalyst, (B) 10Na/Mg_3_AlO_*x*_ sorbent, and (C) ZZO+10Na/Mg_3_AlO_*x*_ catalytic sorbent at 300 °C and atmospheric pressure. The CO_2_ capture was performed by using a 10% CO_2_/N_2_ mixture, and the conversion step was carried out with pure H_2_. Each spectrum corresponds to a 5 min interval.

For 10Na/Mg_3_AlO_*x*_ (Figure 5B), an intense band at 1596 and a shoulder at 1305 cm^–1^, with a Δ*v*_3_ of 291 cm^–1^, could be assigned to bidentate carbonates. However, during the conversion step, the band at 1305 cm^–1^ did not change in intensity. In contrast, the band at 1596 cm^–1^ decreased while shifting toward a lower wavelength of 1561 cm^–1^. On the other hand, the Mg_3_AlO_*x*_ bands at 1572 and 1356 cm^–1^ appeared during the CO_2_ capture step (Figure S7), with a Δ*v*_3_ of 216 cm^–1^. These bands are characteristic of bidentate carbonates.^37^ The intensity of these bands slightly decreased during the conversion step, indicating that they are strongly bound species. Thus, it can be inferred that the band at 1305 cm^–1^ is related to the bidentate carbonates on the Mg–O sites. The intense band at 1596 cm^–1^ might be a combination of the higher-frequency component of the bidentate carbonates and the band related to monodentate carbonates on the Na–O site. Additionally, during the conversion step, low-intensity C–H bands at 2916 and 2843 cm^–1^ appeared; these are related to the CH_4_ formation observed during fixed-bed RCC experiments.

Finally, for the ZZO+10Na/Mg_3_AlO_*x*_ material (Figure 5C), a broad band between 1700 and 1250 cm^–1^ appeared during the CO_2_ capture. This band could be an overlap of the carbonate and HCOO bands formed in the ZZO, the Na sites, and the Mg–O sites. Similarly to the 10Na/Mg_3_AlO_*x*_ sample, upon switching to H_2_ flow during the conversion step, the band at about 1616 cm^–1^ decreased its intensity and shifted to a lower wavenumber. Additionally, small-intensity broad bands at 2801 and 2894 cm^–1^ and small-intensity narrow bands at 2846 and 2916 cm^–1^ appeared during the conversion step. These bands could be related to overlapping peaks of HCOO*, CH_3_O*, and C–H associated with the formation of MeOH and CH_4_ over this material. Based on the differences in the adsorbed species observed by *in situ* DRIFTS, future operando and kinetic experiments at the same elevated pressure conditions as RCC in the fixed bed are needed to fully understand the reaction mechanism(s) during RCC.

### 3.5 Reactive Capture and Conversion

**Figure 6:**
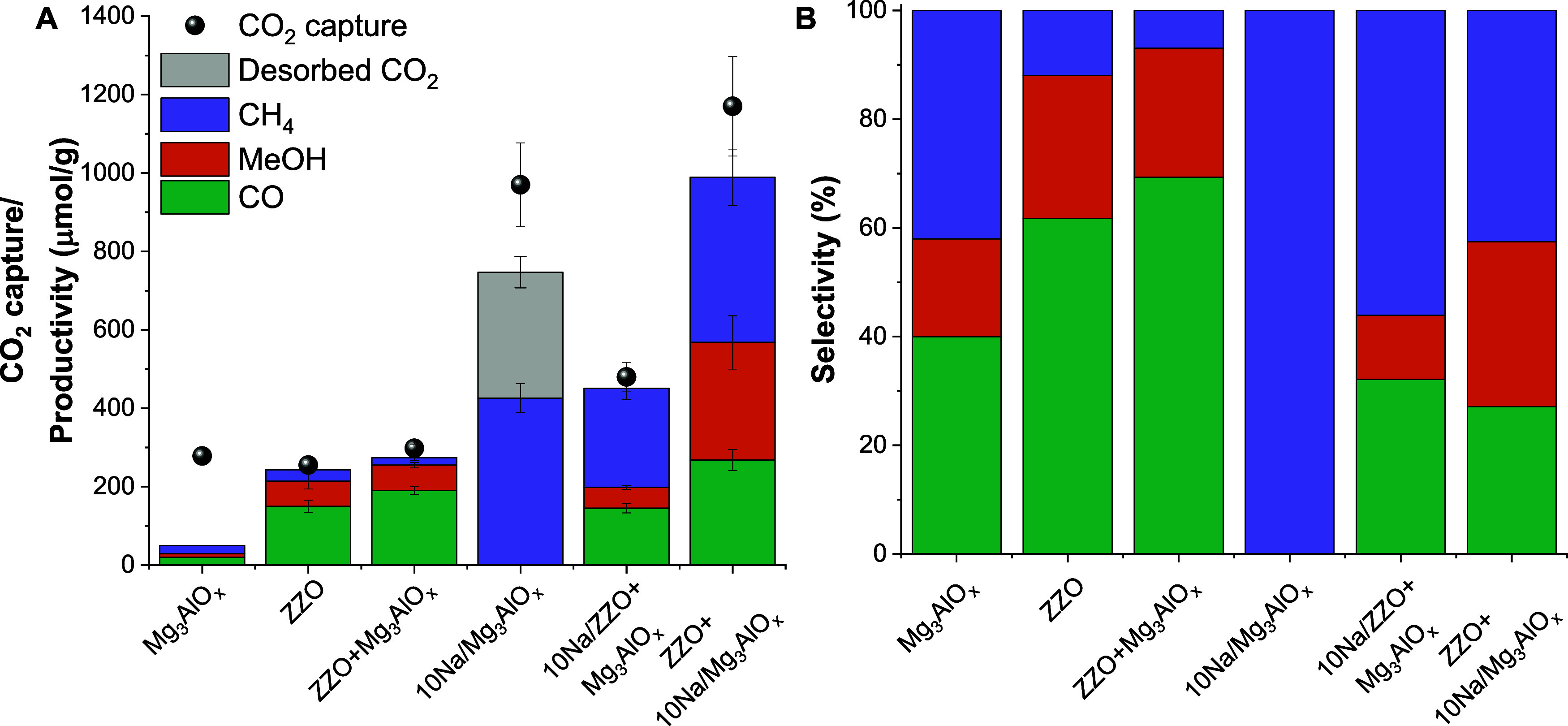
(A) CO_2_ uptake and productivity and (B) product selectivity of different CS after 1 cycle of RCC with CO_2_ capture under 10%CO_2_/N_2_ at 300 °C and conversion under H_2_ at 300 °C and 6 bar.

Figure 6 presents the CO_2_ uptake, productivity, and selectivity to different products in μmol/g_CS_ after 1 cycle of RCC. The adsorption step was performed at 300 °C and atmospheric pressure in 10% CO_2_/N_2_. Total CO_2_ capture was calculated by integrating the breakthrough curves of each CS material and comparing them to the breakthrough curve from a blank SiC bed (Figure S8). Then, the feed was switched to N_2_, and the reactor was purged for 5–7 min. Finally, the feed was switched to H_2_, and the reactor was pressurized to 6 bar. For the CO_2_ capture, N_2_ purge, and H_2_ conversion, a constant flow of 38 mL/min was maintained. In agreement with the observation of CO_2_ uptake by TGA (Section 3.2), Na-impregnated samples had the highest CO_2_ uptakes: 400, 1200, and 970 μmol/g for 10Na/ZZO+Mg_3_AlO_*x*_, ZZO+10Na/Mg_3_AlO_*x*_, and 10Na/Mg_3_AlO_*x*_, respectively. The high CO_2_ uptake for Na/Mg_3_AlO_*x*_ materials may be related to a higher dispersion of Na due to the higher surface area of the Mg_3_AlO_*x*_ support as opposed to the ZZO catalyst support (Table 1).

Without ZZO catalyst, no MeOH formation was observed, and only small amounts of CH_4_ and CO were detected, which are the most thermodynamically favorable products under these conditions, with Δ*G*°= −130.8 and −29 kJ/mol, respectively. On the contrary, for ZZO and ZZO+Mg_3_AlO_*x*_, the CO_2_ conversion was about 90%, and CO and MeOH were the main products detected. Similar to the steady-state cofeed experiments (Section 3.3), the addition of Mg_3_AlO_*x*_ had no effect on the activity of the ZZO catalyst, and the product distribution was similar for both materials (Figure 6B). Interestingly, for all of the Na-impregnated materials, CH_4_ was detected as the major product; in the case of 10Na/Mg_3_AlO_*x*_, unreacted CO_2_ was also detected during the conversion stage, and the CO_2_ conversion was only 40%. For the 10Na/ZZO+Mg_3_AlO_*x*_, the CO_2_ conversion was about 84% and the MeOH productivity was 50 μmol/g, which corresponds to a selectivity of 11%, while for the ZZO+10Na/Mg_3_AlO_*x*_ material, the CO_2_ conversion was lower (84%), but the MeOH productivity and selectivity were higher, 300 μmol/g and 30%, respectively. As observed during steady-state cofeed experiments, direct deposition of Na on ZZO yielded the lowest catalytic activity, which could also explain the low selectivity for MeOH over the 10Na/ZZO+Mg_3_AlO_*x*_.

The observed CH_4_ formation during RCC might be related to the reaction conditions, specifically, moderate temperature (300 °C) and excess H_2_. At moderate temperatures (300–400 °C), CH_4_ is favored (relative to low-temperature methanol synthesis) because the reaction involves an endothermic step to produce CO, which is accepted in the literature as a key reaction intermediate.^38^ While CO_2_ methanation is an exothermic reaction (), moderate temperatures are necessary to facilitate the endothermic CO formation while maintaining favorable conditions for CH_4_ formation. Moreover, the presence of excess H_2_ may promote CH_4_ formation, as the stoichiometric H_2_:CO_2_ ratio for CH_4_ production (4:1) is higher than the required for CH_3_OH synthesis (3:1). However, during steady-state cofeed experiments over ZZO+10Na/Mg_3_AlO_*x*_ in excess H_2_ (Figure S9), only MeOH and CO were detected. The absence of CH_4_ under similar conditions in the cofeed experiments suggests the involvement of additional factors influencing the product distribution during RCC. To better understand this complexity, the effects of CS configuration, temperature, and pressure on RCC product distribution were evaluated.

**Figure 7:**
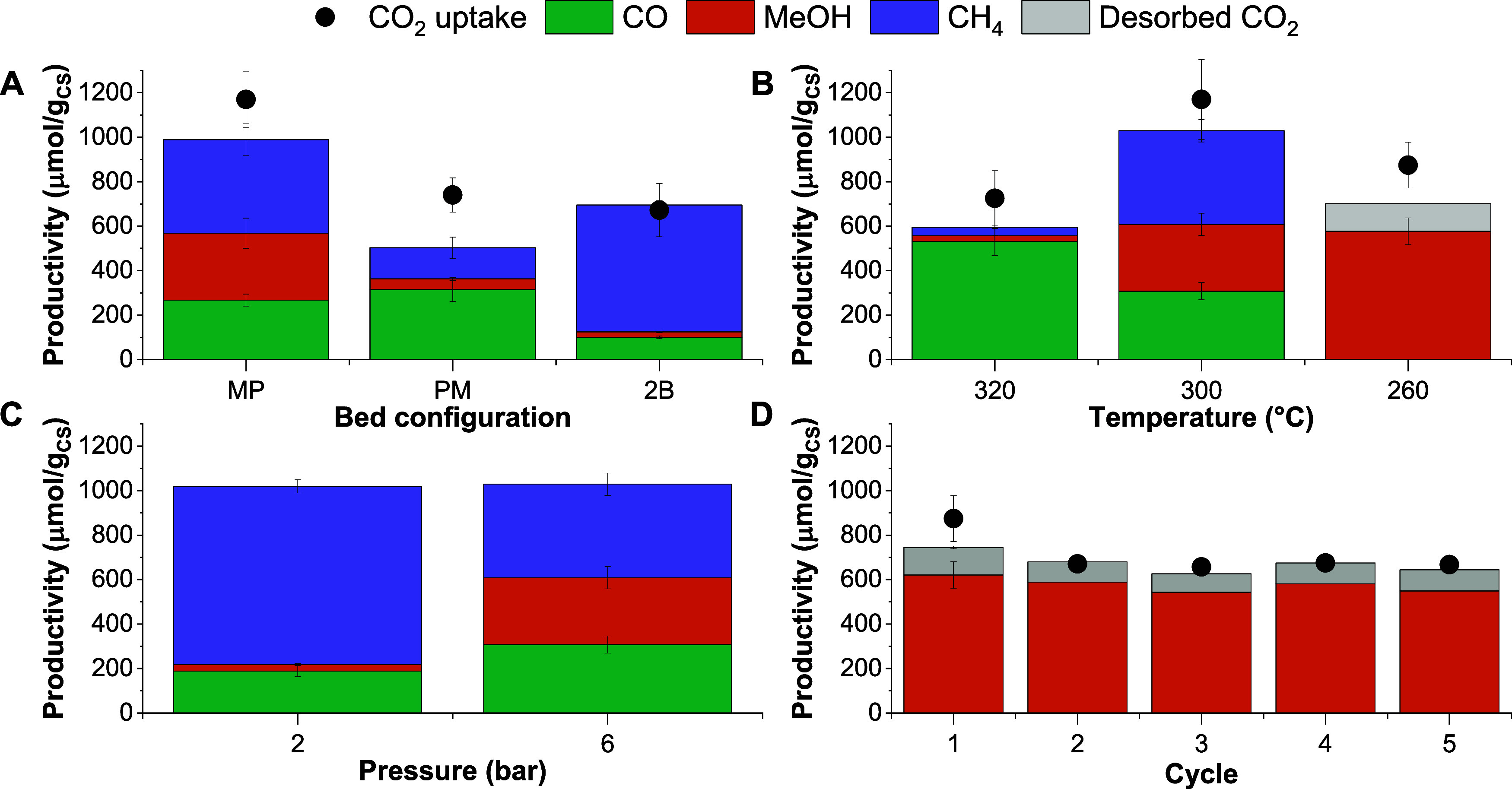
CO_2_ uptake and productivity during RCC over ZZO+10Na/Mg_3_AlO_*x*_ for (A) different CS configurations, (B) different temperatures, (C) different pressures at 300 °C, and (D) 5 consecutive cycles of RCC at 260 °C for the MP configuration.

Figure 7A shows the CO_2_ uptake and product distribution for different CS sorbent configurations, including (i) mixed and pelletized (MP), in which ZZO and 10Na/Mg_3_AlO_*x*_ were mixed and ground, then pelletized together, (ii) pelletized and mixed (PM), in which ZZO and 10Na/Mg_3_AlO_*x*_ were pelletized separately and then mixed before placing the mixture in the reactor, and (iii) 2 beds in which 10Na/Mg_3_AlO_*x*_ pellets and ZZO pellets were placed in the reactor separated by a quartz wool bed. The CO_2_ uptake and productivity increased when transitioning from no contact to intimate contact between the catalyst and the sorbent, with MP > PM > 2-bed. Moreover, the MeOH selectivity also increased from 3 to 30% from the 2-bed configuration to the MP sample; this suggests that intimate contact between the catalyst and sorbent can modulate the product distribution and formation of MeOH.

Figure 7B shows the CO_2_ uptake and product distribution for different RCC temperatures ranging from 260 to 320 °C. CO_2_ uptake had a volcano-type relationship with temperature, with a maximum at 300 °C (1170 μmol/g), this behavior has also been reported by other authors for Na(NO_3_/NO_2_) promoted MgO and Mg_*x*_Al_*y*_Ox mixed oxides.^21,39^ For 300 and 320 °C, CH_4_ and CO formation were predominant, with selectivities of 40 and 29% and 33 and 62%, respectively. RWGS is an endothermic reaction; thus, it is favored at higher temperatures, consistent with the high selectivity of CO obtained for the RCC performed at 320 °C. At 260 °C, the CO_2_ conversion decreased and unreacted CO_2_ desorption was also detected during the conversion stage. At this temperature, MeOH selectivity was 100% with the highest productivity, about 580 μmol/g/cycle, among the conditions tested. Additionally, 5 consecutive RCC cycles were performed at 260 °C (Figure 7C). The initial CO_2_ uptake of 874 μmol/g slightly decreased to about 650 μmol/g for the second cycle. However, The MeOH productivity remained stable at around 580 μmol/g over the 5 consecutive cycles.

**Figure 8:**
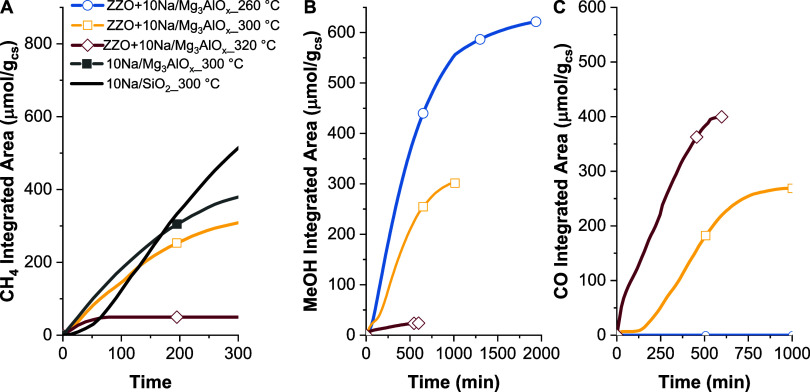
Rate of (A) CH_4_, (B) MeOH, and (C) CO production during the conversion step for ZZO+10Na/Mg_3_AlO_*x*_, 10Na/Mg_3_AlO_*x*_, and 10Na/SiO_2_, obtained from integration of the product flow rate quantified by GC.

In Figure 8A, it is observed that the CH_4_ formation rate has almost no variation for the different temperatures over the ZZO+10Na/Mg_3_AlO_*x*_ CS, except at 260 °C, where it was not detected. The initial CH_4_ formation rate is similar for the 10Na/Mg_3_AlO_*x*_ material at 300 °C (gray line in Figure 8A), even in the absence of the ZZO catalyst. On the contrary, for MeOH (Figure 8B) and CO (Figure 8C), there is variation in the rate with respect to temperature, a trend also observed in steady-state cofeed experiments at different temperatures (Figure S10). Moreover, with increasing temperature, the time to complete the reaction decreased from about 1200 min at 260 °C to about 600 min at 320 °C. Additionally, for MeOH and CO synthesis during RCC, an induction period was observed (Figures 8B,C and S11). These observations suggest that CH_4_ is synthesized from a similar intermediate adsorbed on a Na active site under the RCC conditions. *In situ* DRIFTS analysis (Section 3.4) evidence the formation of different carbonate species on the Na-containing materials as opposed to Na-free compositions.

**Figure 9:**
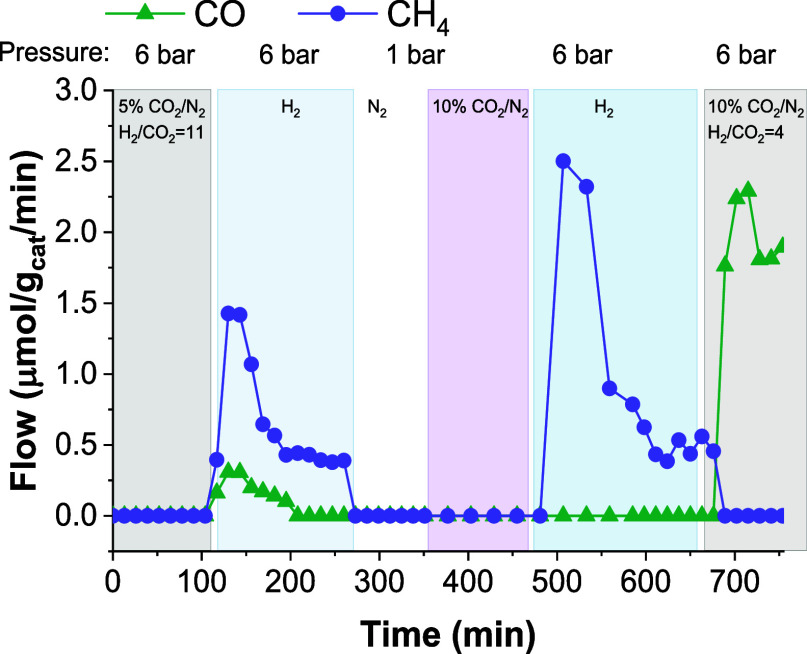
Product formation variations between cofeed steady-state and RCC conditions over 10Na/Mg_3_AlO_*x*_ at 300 °C.

Figure 9 presents changes in product species during fixed-bed experiments over 10Na/Mg_3_AlO_*x*_ when switching between steady-state cofeed and RCC conditions, reflecting different CO_2_ surface coverages and H_2_ partial pressures. First, for low CO_2_ coverage and high H_2_ partial pressure (i.e., switch between cofeed with 5% CO_2_/N_2_ and pure H_2_ at 6 bar, both CH_4_ and CO were detected. Then, for high CO_2_ coverage and high H_2_ partial pressure (RCC conditions) only CH_4_ was detected. Lastly, for moderate CO_2_ coverage and moderate H_2_ partial pressure (i.e., switch between partially CO_3_-depleted material and cofeed conditions with 40% H_2_ at 6 bar) only CO was detected. This suggests that under RCC conditions, evolution of CO_2_ and H_2_ surface coverage (as driven by H_2_ partial pressure), modulate the product distribution, with CH_4_ being favored at high CO_2_ coverages at the initial stages of the conversion step and then transitioning to CO and MeOH, at lower CO_2_ coverages. This rationalizes the induction period observed for the CO and MeOH in Figure 8. A similar behavior was reported by Shi et al. when studying CaCO_3_ decomposition under varying H_2_ partial pressures (P_H2_) from (1 – 60 bar). At low P_H2_, CO formation was favored, while at high P_H2_, CH_4_ was favored.^40^

We also considered that some Na precursors, such as Na_2_CO_3_, have been previously reported to contain low levels of metal impurities, such Pd, in the range of ppb, leading to erroneous claims of “metal-free” catalysis.^41^ Thus, we surmised that the formation of CH_4_ over Na/Mg_3_AlO_*x*_ could be related to the presence of low levels of metal contamination. However, neither Pd nor other unexpected metals were detected in our metal-free compositions. Furthermore, CH_4_ is observed exclusively under RCC conditions and not during steady-state cofeed conditions. This indicates that the transient conditions and evolving catalyst surface coverages play a dominant role in the product distribution during RCC and enable reactions that are not observed during cofeed steady-state conditions.

Previous reports using the RCC approach for MeOH synthesis have explored various catalytic sorbent configurations and composition. Wirner et al. utilized a 2-bed configuration consisting of a CZA catalyst combined with 16 wt % Na/Al_2_O_3_ in a temperature-swing process, with CO_2_ capture at 160 °C and 0.8 bar and conversion at 260 °C and 9 bar.^8^ The authors reported significant formation of CO (70% selectivity) and less CH_4_ (10% selectivity). A further decrease in MeOH selectivity was observed when the CZA catalyst was placed in direct contact with the Na/Al_2_O_3_. They attributed this behavior to the oxidation of the CZA catalyst during the CO_2_ capture phase, but it could also be related to higher Na content (16 wt %) in their system, which likely contributed to more severe catalyst deactivation through site poisoning. This resulted in a MeOH productivity of 12 μmol/g and high unreacted CO_2_ desorption (150 μmol/g), indicating low CO_2_ conversion, which could be related to the bed configuration that the authors used. Because Wirner et al. did not report the total CO_2_ uptake, this limits a full comparison of the CO_2_ capture efficiency and conversion between that system and this current work.

Lastly, Potter et al. explored using 5 wt % K- and Ca-impregnated CZA catalyst as a CS, also in a temperature-swing process, with CO_2_ capture at 100 °C and 0.8 bar and conversion at 250 °C and 30 bar.^2^ They reported CO_2_ uptakes of 150 μmol/g for K and 70 μmol/g for Ca materials, with methanol productivities of 50 and 20 μmol/g, respectively. The authors attributed the superior performance of the K-impregnated catalyst to its higher CO_2_ uptake and a closer proximity between the adsorbed CO_2_ species and the H_2_ activation sites due to direct impregnation. Although this approach improved MeOH productivity compared to that of Wirner et al., the potential for further enhancing CO_2_ uptake by increasing the alkali loading is constrained by the risk of catalyst deactivation, which ultimately limits CS MeOH productivity.

For the ZZO+10Na/Mg_3_AlO_*x*_ CS in the current work, higher CO_2_ uptake (1100 μmol/g) and MeOH productivity (300 μmol/g) were obtained at 300 °C and 6 bar, respectively. Consistent with Potter et al., this superior performance can be attributed to the synergy between the sorbent and catalyst, achieved by placing the two materials in close contact without sacrificing catalytic activity through direct alkali impregnation. As opposed to previous reports, which employed a temperature-swing process, here, RCC was evaluated under isothermal conditions. This approach helps maintain consistent reaction conditions, potentially reducing desorption of CO_2_ losses and enabling higher methanol productivity. Additionally, the limitations reported in previously studied CS can be overcome by moderating catalyst poisoning through alkali promoter impregnation on a different support. In our case, a Mg-based sorbent support with a high BET surface area was employed, which allowed for better dispersion of Na. Moreover, the interaction between Na and Mg in Mg_3_AlO_*x*_ creates new CO_2_ adsorption sites.

Given this enhanced CO_2_ uptake and MeOH productivity, it is important to consider how the observed minor improvements impact the overall feasibility of RCC, particularly in terms of economic viability and scalability. Martin et al.^42^ reported preliminary results of techno-economic analysis and a life cycle assessment for RCC and compared it with the traditional baseline scenario in which CO_2_ is captured, purified, and then fed to a hydrogenation reactor for its conversion to MeOH. The authors employed a CZA catalyst with a total CO_2_ uptake of 66 μmol/g and a MeOH productivity of 15 μmol/g for the RCC approach. With this performance, they found that RCC had a 3 times higher MeOH levelized cost than the baseline scenario without recycling of unreacted species. This high cost was driven mostly by the production of H_2_ from water electrolysis. With the MeOH productivity of 15 μmol/g for the CZA, the authors reported a high H_2_:MeOH ratio of 1 g-H_2_/g-MeOH, and the authors estimated that at 0.2 g-H_2_/g-MeOH would be needed for the MeOH levelized cost of RCC to be lower than the baseline scenario. In contrast, the ZZO+10Na/Mg_3_AlOx catalytic sorbent explored in this study exhibited a higher methanol productivity of 580 μmol/g, which could lead to a lower H_2_:MeOH ratio, closer to the target estimated by Martin et al.^42^ However, the feasibility of RCC for the synthesis of MeOH from CO_2_ remains challenged. For example, the slow observed kinetics of the conversion step, with the reaction requiring about 1200 min to be completed at 260 °C, present a challenge. This is equivalent to a normalized MeOH productivity of 0.5 μmol/g/min. This performance is higher than that reported by Potter et al.^2^ (0.3 μmol/g/min), yet the process conditions vary considerably. Potter et al. used a pressure- and temperature-swing process at 30 bar, in contrast to the isothermal approach at 6 bar employed in this study. Thus, further studies are needed to optimize reaction conditions, taking into account the energy consumption, MeOH productivity, and CO_2_ emissions.

**Figure 10:**
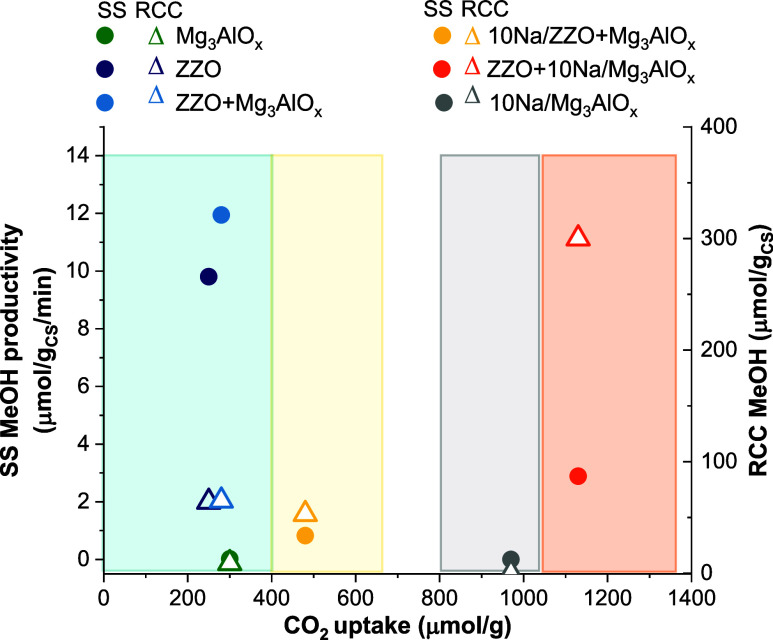
Steady-state cofeed MeOH productivity and RCC MeOH productivity as a function of CO_2_ uptake for all of the sorbent, catalyst, and CS samples tested.

As a summary, Figure 10 presents the steady-state cofeed MeOH productivity and RCC MeOH productivity as a function of the CO_2_ uptake obtained for the different materials evaluated here. It can be observed that the highest MeOH productivity was obtained for the ZZO+10Na/Mg_3_AlO_*x*_ catalytic sorbent, in which a balance between the catalytic activity and total CO_2_ uptake was achieved. High MeOH cofeed STY and sufficient CO_2_ uptake are required for high RCC MeOH activity. For example, the ZZO and ZZO+ Mg_3_AlO_*x*_ had the highest steady-state cofeed activity, but low CO_2_ uptake, resulting in low RCC MeOH productivity. Enhancing the CO_2_ uptake with Na impregnation on the ZZO, rising from 270 to 480 μmol/g, resulted in poisoning of the catalyst and low cofeed MeOH STY, resulting in low MeOH productivity. On the other hand, the 10Na/Mg_3_AlO_*x*_ had a higher CO_2_ uptake (970 μmol/g), but without a MeOH synthesis catalyst, no MeOH was obtained during RCC. Thus, the ZZO mixed with 10Na/Mg_3_AlO_*x*_ offers a balance between moderate catalyst poisoning and enhanced CO_2_ uptake that results in a high MeOH productivity during RCC.

## 4 Conclusions

Good CO_2_ uptake (1100 μmol/g) and MeOH productivity were obtained with a ZnZrO_2_+10Na/Mg_3_AlO_*x*_ CS (300 μmol/g) at 300 °C and 6 bar, which are higher than previous reports of CZA catalytic sorbents. It was observed that the CO_2_ uptake and MeOH selectivity were strongly influenced by the sorbent configuration, temperature, and pressure, with a clear balance required between the CO_2_ adsorption capacity and catalytic activity to generate high RCC MeOH productivity. Alkali impregnation on the Mg-based sorbent moderated catalyst poisoning, allowing for improved CO_2_ capture and conversion without compromising the catalytic activity. Under the explored reaction conditions, operation at 260 °C resulted in the highest MeOH productivity of 580 μmol/g. However, a large amount of unreacted CO_2_ was also detected, indicating a low conversion. Additionally, at 260 °C, the conversion step requires over 1000 min to be completed. Thus, further studies are needed to optimize both MeOH selectivity and overall conversion, as well as to improve reaction rates.

Additionally, the significance of the transient conditions during RCC was demonstrated, with such conditions favoring reactions that are not observed under steady-state cofeed conditions. Steady-state catalysis conditions can, thus, not be used to easily and directly inform transient RCC performance for this system. *In situ* DRIFTS revealed different carbonate species formed over Na-impregnated materials as opposed to the ZnZrO_2_ catalyst, and multiple species were detected for the ZZO+10Na/Mg_3_AlO_*x*_ CS. However, it is not yet clear whether these distinct species are active intermediates contributing to different products or are simply spectators. To deconvolute this effect, further kinetic and operando spectroscopy experiments are to be pursued in future work.

## Supporting Information Available

Detailed material synthesis; material characterization including N_2_ isotherms, combustion TGA, H_2_-TPR, and STEM-EDS; time evolution of DRIFTS band intensity; CO_2_ breakthrough curves; additional reaction conditions for cofeed experiments; and the concentration evolution of RCC for different tested materials (PDF)
