# Supplementary material for: Reactive Capture and Conversion of Carbon Dioxide to Methanol with ZnZrO2 and Alkali-Promoted Mg3AlOx Mixed Oxide Catalytic Sorbents
Source: ACS Sustain Chem Eng. 2025 Mar 21;13(12):4811–22. doi: 10.1021/acssuschemeng.4c10562 (PMC11962838; doi:10.1021/acssuschemeng.4c10562)
Supplement: Supplementary file 1 — sc4c10562_si_001.pdf [file sc4c10562_si_001.pdf]

Supporting Information for:

Reactive Capture and Conversion of Carbon Dioxide to Methanol with $ZnZrO_2$ and Alkali -Promoted Mg_3-Al(Ox) Mixed Oxide Catalytic Sorbents

Laura Proaño[1], Katlo Galefete[1], Guanhe Rim[1], Gabriel Gusmão[1] and Christopher W. Jones[1]*

[1] School of Chemical & Biomolecular Engineering, Georgia Institute of Technology, Atlanta, GA 30332 (United States)

*Corresponding author: cjones@chbe.gatech.edu

Number of pages: 9

Number of figures: 10

Number of tables: 1

Table of Contents

Detailed material synthesiss1

Materials characterizations1

Figure S1. Combustion TGA and DTG of the synthesized Mg_3Al-CO_3 hydrotalcite.................S3

Figure S2. N_2 isotherms of (A) fresh Mg_3AlO_x, calcined and 10% $NaNO_3$-impregnated hydrotalcites and (B) the $ZnZrO_2$ catalyst and $ZnZrO_2$+Mg_3AlO_x catalytic sorbents..................S3

Figure S3. STEM-EDS images of $ZnZrO_2$+Mg_3AlOx..S4

Figure S4. H_2-TPR profiles of the $ZnZrO_2$ catalyst (blue), Mg_3AlO_x sorbent, and physical mixture of $ZnZrO_2$ and Mg_3AlOx sorbent. S4

Figure S5. Observed and calculated rates of MeOH (left) and CO (right) for (A) ZZO, (B) ZZO+10Na/Mg_3AlO_x, and (C) 10Na/ZZO+ Mg_3AlO_x . Alpha and beta indicate the apparent reaction orders of CO_2 and H_2, respectively, obtained from the non-linear fitting conducted in MATLAB, to the power law in equation ES1. ....S5

Figure S6. Time evolution of band intensity for (A) peaks in the region between 1000 – 1700 cm^{-1} and (B) peaks in the region between 2800 – 3000 cm^{-1} during *in situ* DRIFTS RCC performed at atmospheric pressure and 300 °C over the $ZnZrO_2$ catalyst...S6

Figure S7. *In situ* DRIFTS spectra during CO_2 capture and conversion over the Mg_3AlOx sorbent at 300 °C and atmospheric pressure. The CO_2 capture was performed using a 10% CO_2/N_2 mixture, and the conversion step was carried out with pure H_2..S7

Figure S8. CO_2 breakthrough curves obtained for the different CS. S7

Figure S9. MeOH selectivity and CO_2 conversion over ZZO+10Na/Mg_3AlO_x catalytic sorbent for varying H_2:CO_2 ratios. S8

Figure S10. Co-feed steady-state CO_2 conversion and MeOH selectivity for different CS at different temperatures, pressures and WHSV. S8

Figure S11. Rate of (A) CH_4, (B) MeOH, and (C) CO production during the conversion step at 300 °C and 6 bar for all the catalytic sorbents..S9

Figure S12. Flow profiles obtained during the conversion step of RCC for (A) Mg_3AlO_x, (B) ZZO, (C) ZZO+ Mg_3AlO_x, (D) 10Na/ZZO+ Mg_3AlO_x , and (D) ZZO+10Na/ Mg_3AlO_x at 300 °C and 6 bar. Each profile is the average of 2 different experiments..S9

Figure S13. Flow profiles obtained during the conversion step of RCC for ZZO+10Na/ Mg_3AlO_x at 300 °C and 6 bar for (A) 2-bed configuration, (B) pelletized and mixed configuration and (C) the mixed and pelletized configuration at 320 °C and (D) the mixed and pelletized configuration at 260 °C . Each profile is the average of 2 different experiments..S10

Detailed material synthesis

1. $ZnZrO_2$ catalysts

To start, 0.6 g of $Zn(NO_3)_2 \cdot 6H_2O$ and 5.8 g of $Zr(NO_3)_4 \cdot 5H_2O$ were dissolved in 100 mL of deionized water to form solution A. Separately, 3.06 g of $(NH_4)_2CO_3$ was dissolved in 100 mL of deionized water and added to solution A at 10 mL/min using a peristaltic pump at 70 °C under vigorous stirring (400 rpm). The resulting suspension was aged for 2 hours at constant temperature and stirring, then filtered, washed with deionized water, dried overnight at 110 °C, and calcined at 500 °C for 4 hours in static air.

2. Mg_3AlOx sorbent

A 1 M aqueous solution (100 mL) of $Mg(NO_3)_2 \cdot 6H_2O$ and $Al(NO_3)_3 \cdot 9H_2O$ with a Mg:Al ratio of 3:1 was slowly added to 100 mL of 0.5 M Na_2CO_3. The pH was maintained at 10.0 ± 0.2 during synthesis using 4 M NaOH. After aging at room temperature for 24 hours, the precipitate was washed with deionized water until the pH reached 7.0, re-dispersed in acetone, and aged for 30 minutes. The material was then filtered, vacuum dried at 60 °C, and calcined in situ at 400 °C to produce Mg_3AlO_x mixed metal oxide support.

Materials characterization

The Brunauer-Emmett-Teller (BET) surface area of the calcined supports was determined by N_2 physisorption performed on a Tristar II 3020 (Micromeritics). at 77 K using ~200 mg of sorbent. Samples were degassed under vacuum at 150 °C for 3 h prior to measurements.

X-ray Powder diffraction (XRD) patterns of the as-prepared $ZnZrO_2$ catalyst, Mg_3Al-CO_3 hydrotalcite, and alkali-impregnated catalytic sorbents were recorded at room temperature on a Rigaku diffractometer using Cu Kα radiation in the 2θ range from 20° to 60°.

Scanning transmission electron microscopy (STEM) and energy dispersive X-ray spectroscopy (EDS) analysis were conducted on an aberration-corrected Hitachi HD2700, equipped with a Bruker SDD EDS detector. Samples were prepared by sonicating the catalyst in isopropyl alcohol, then adding a few drops of the solution onto holey carbon-coated Cu grids.

CO_2 temperature programmed desorption (CO_2-TPD) and H_2 temperature programmed reduction (H_2-TPR) were performed using an Autochem II 2920 (Micromeritics, USA) equipped with a TCD detector. For CO_2-TPD, approximately 100 mg of powder catalyst was loaded into a U-shaped quartz tube, reduced at 400 °C for 2 hours in 10% H_2/Ar, and then treated with CO_2 for 60 minutes. The catalyst was flushed with He at 40 °C until a stable baseline was achieved, followed by heating from 40 to 800 °C at 5 °C/min in He, with CO_2 desorption monitored by the TCD. For H_2-TPR, the calcined catalyst was loaded into a U-shaped quartz tube, pre-treated in He at 150 °C for 2 hours, cooled to 40 °C, and then reduced in 10% H_2/Ar while heating at 5 °C/min to 800 °C.

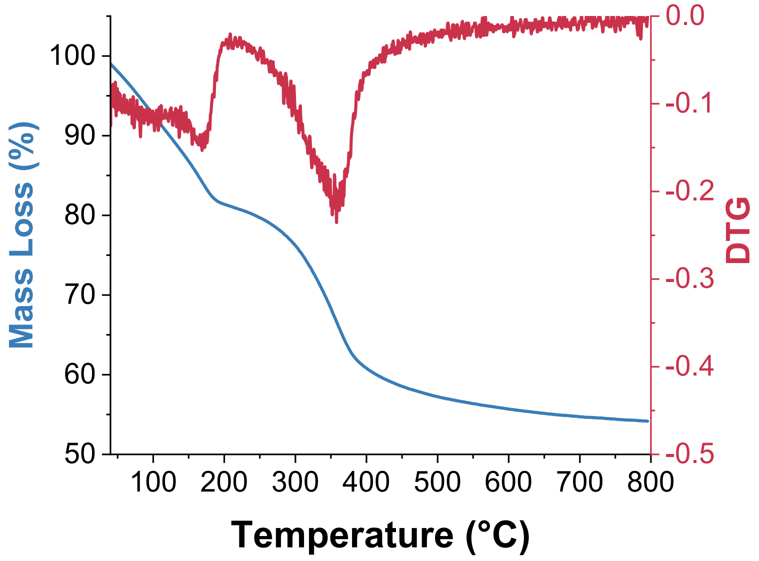

Figure S1. Combustion TGA and DTG of the synthesized $Mg_3Al\text{-}CO_3$ hydrotalcite.

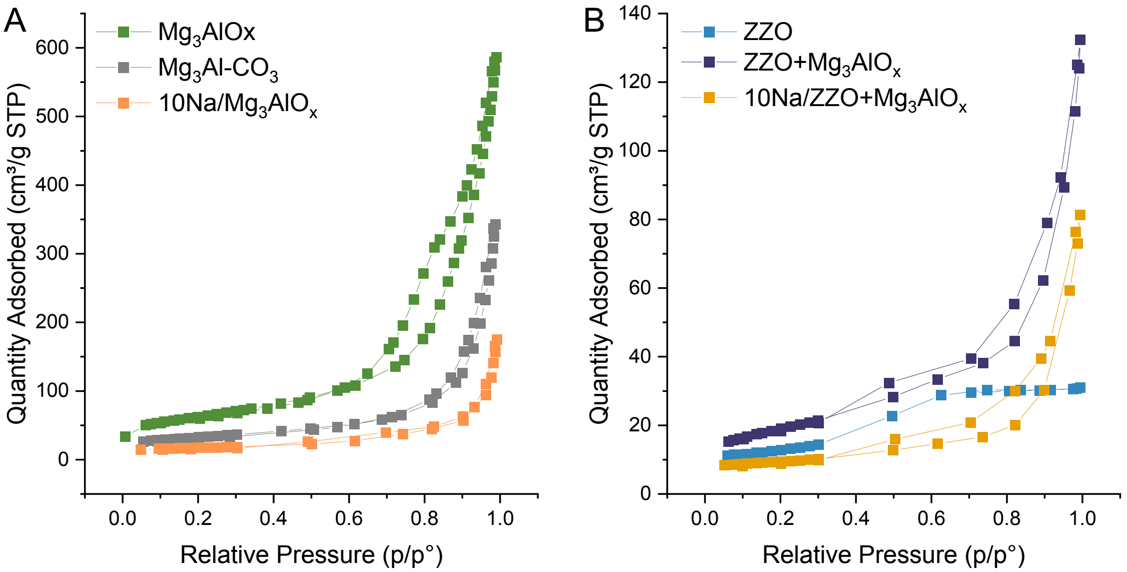

Figure S2. N_2 isotherms of (A) fresh Mg_3AlO_x, calcined and 10% $NaNO_3$-impregnated hydrotalcites and (B) the $ZnZrO_2$ catalyst and $ZnZrO_2$+Mg_3AlO_x catalytic sorbents.

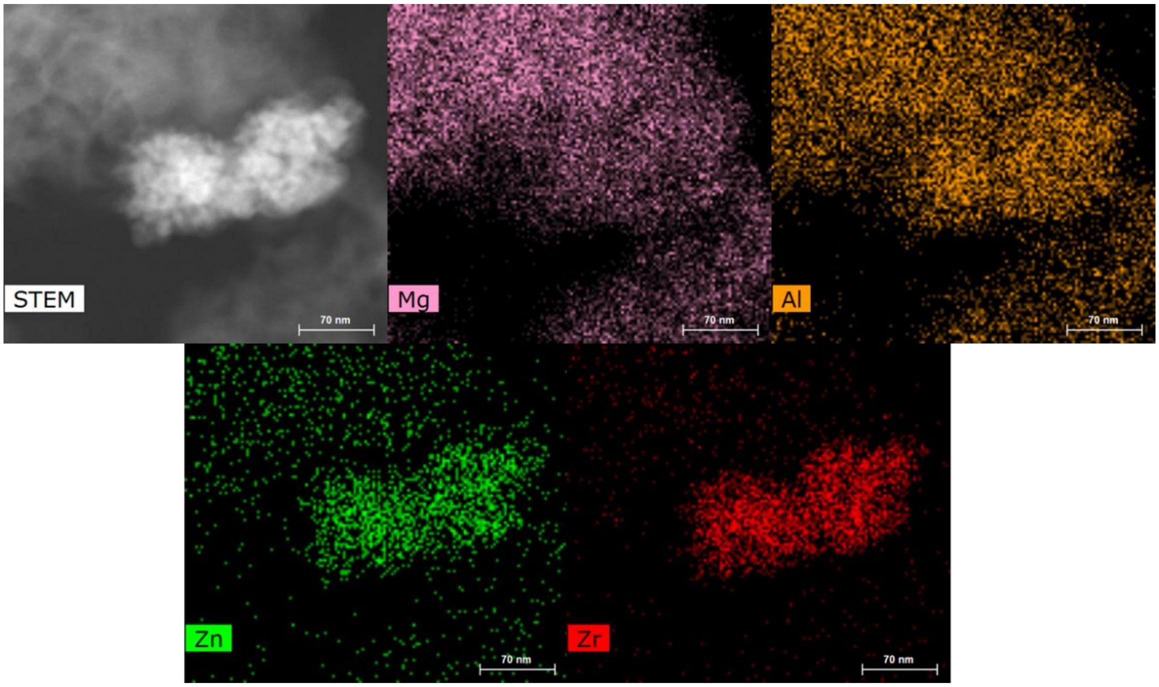

Figure S3. STEM-EDS images of $ZnZrO_2$+Mg_3AlOx

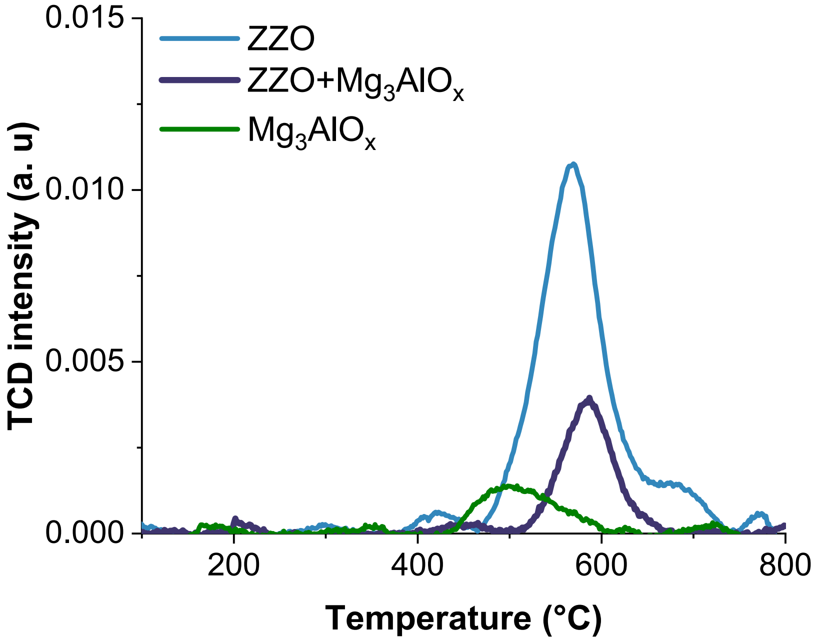

Figure S4. H_2-TPR profiles of the $ZnZrO_2$ catalyst (blue), Mg_3AlO_x sorbent, and physical mixture of $ZnZrO_2$ and Mg_3AlOx sorbent.

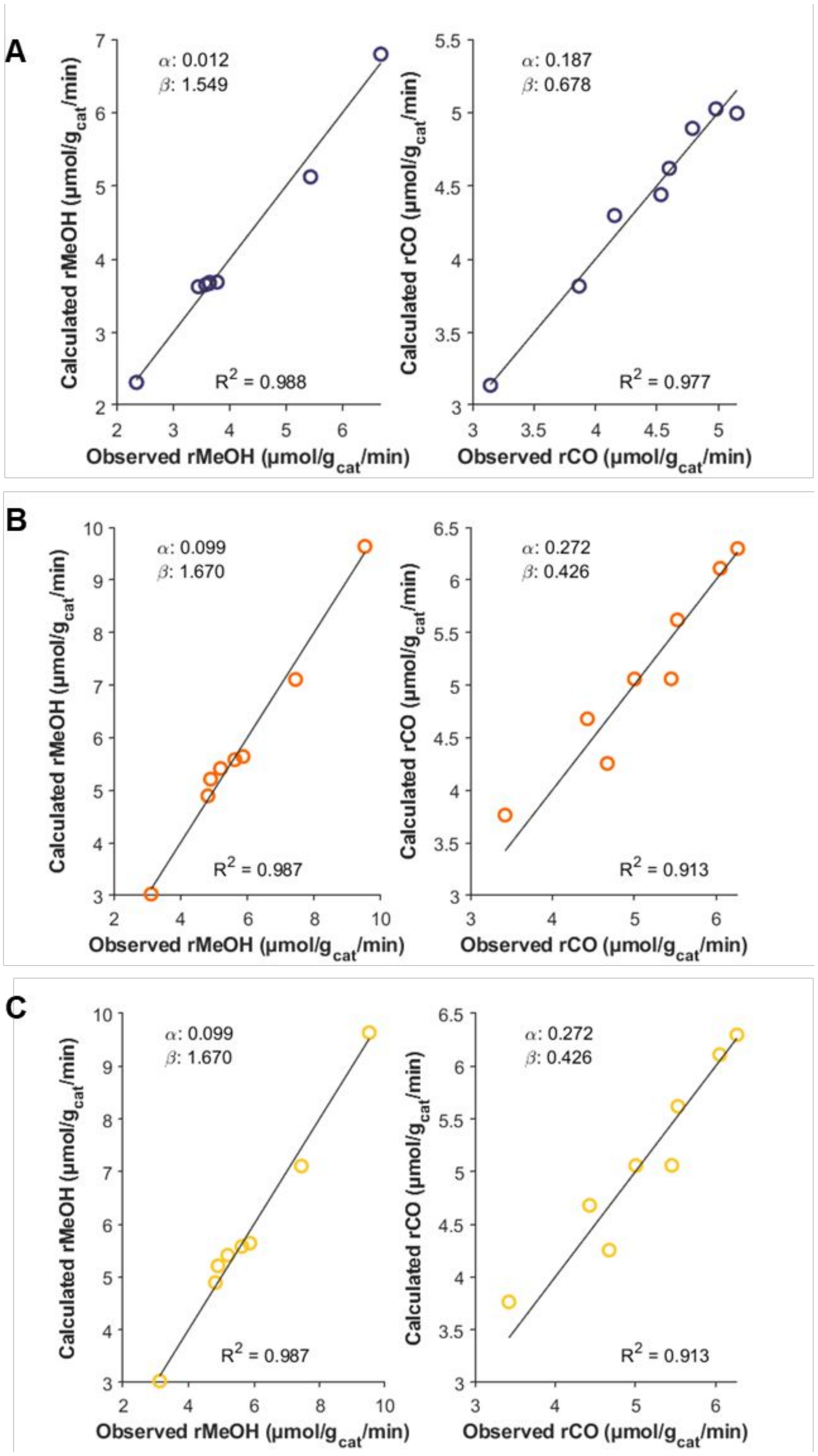

Figure S5. Observed and calculated rates of MeOH (left) and CO (right) for (A) ZZO, (B) ZZO+10Na/Mg_3AlO_x, and (C) 10Na/ZZO+ Mg_3AlO_x . Alpha and beta indicate the apparent reaction orders of CO_2 and H_2, respectively, obtained from the non-linear fitting conducted in MATLAB, to the power law in equation ES1.

$$\ln(r_i) = \ln(k_i) + \alpha\ln(P_{CO2}) + \beta\ln(P_{H2}) \quad \text{(ES1)}$$

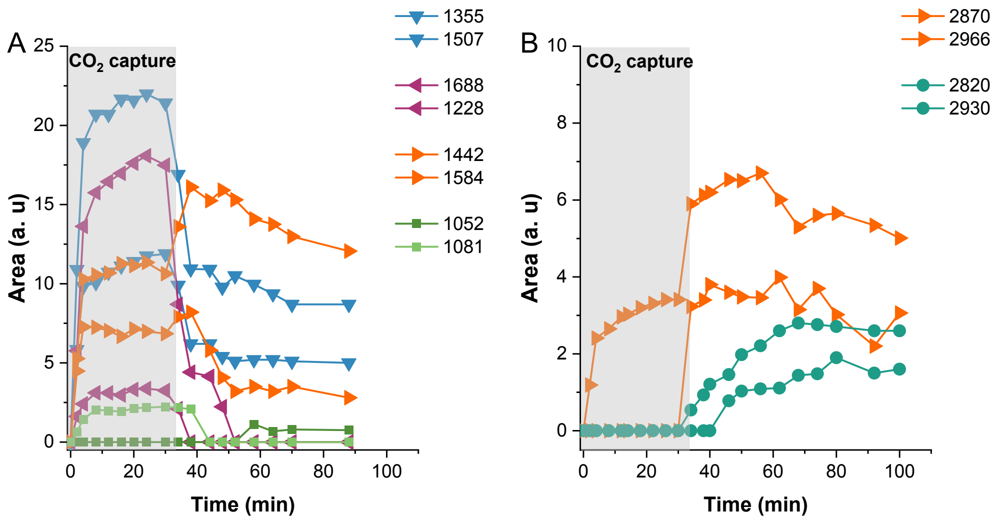

Figure S6. Time evolution of band intensity for (A) peaks in the region between 1000 – 1700 cm^{-1} and (B) peaks in the region between 2800 – 3000 cm^{-1} during *in situ* DRIFTS RCC performed at atmospheric pressure and 300 °C over the $ZnZrO_2$ catalyst.

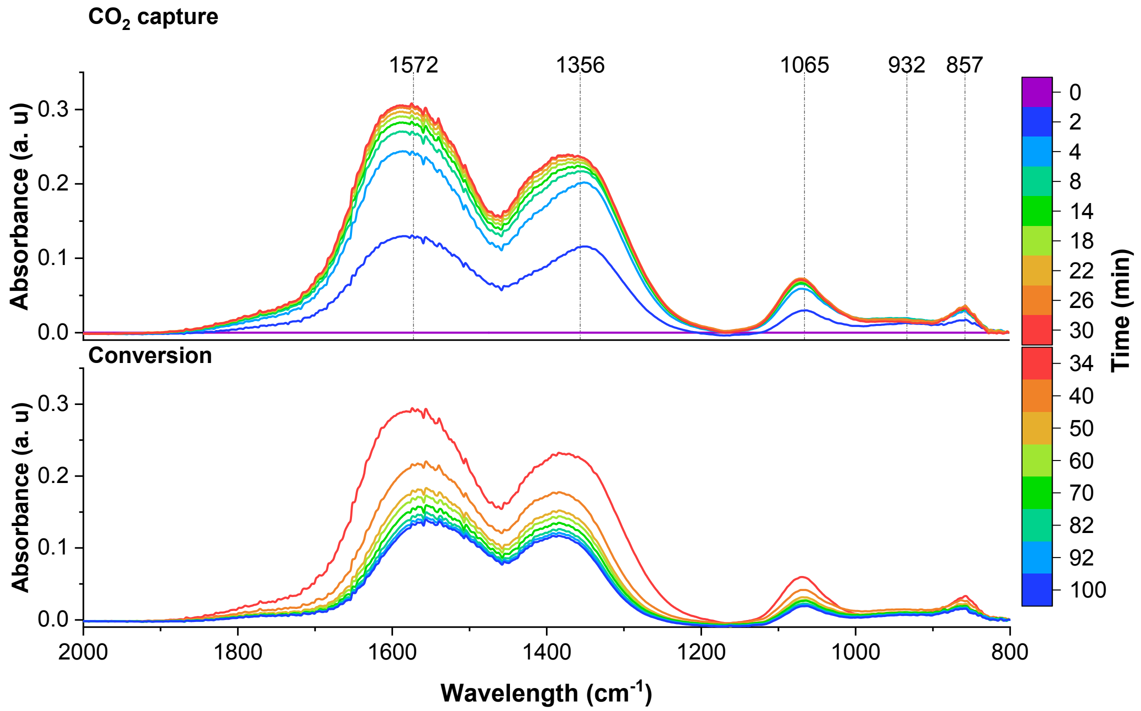

Figure S7. *In situ* DRIFTS spectra during CO_2 capture and conversion over the Mg_3AlOx sorbent at 300 °C and atmospheric pressure. The CO_2 capture was performed using a 10% CO_2/N_2 mixture, and the conversion step was carried out with pure H_2.

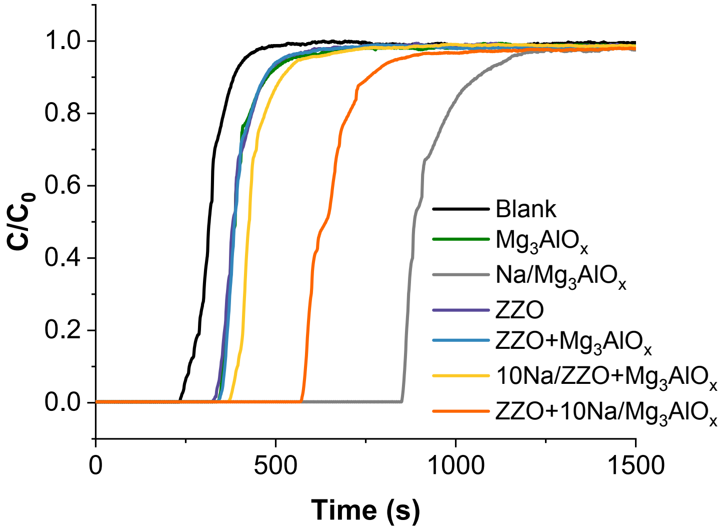

Figure S8. CO_2 breakthrough curves obtained for the different CS.

CO_2 uptake was calculated from equation S1, where $\dot{V}$ is the flow rate in mL/s, y_{CO2} is the CO_2 concentration, C_t is the concentration of CO_2 recorded by Quantek IR gas analyzer (Model 906) and C_0 is the concentration of CO_2 in the feed gas stream and $C_{t,blank}$ is the recorded CO_2 concentration at time *t* for a SiC filled reactor.

$$q\left(\frac{mmol}{g}\right) = \frac{\dot{V} y_{CO2}}{m}\int_0^{ts}\left(\frac{C_t}{C_0} - \frac{C_{t,\,blank}}{C_0}\right) \qquad \text{(S1)}$$

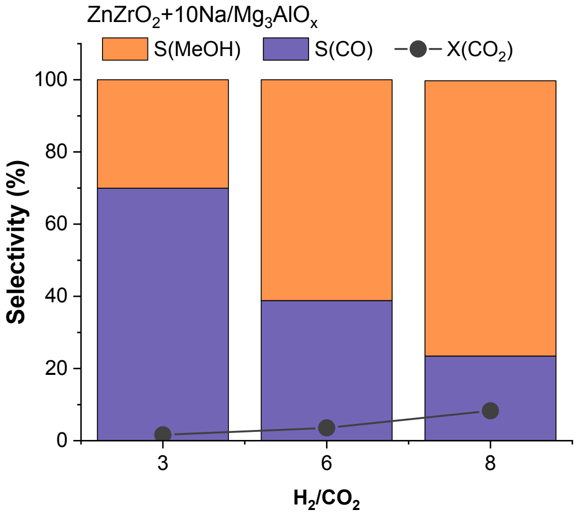

Figure S9. MeOH selectivity and CO_2 conversion over ZZO+10Na/Mg_3AlO_x catalytic sorbent for varying H_2:CO_2 ratios during co-feed steady-state conditions.

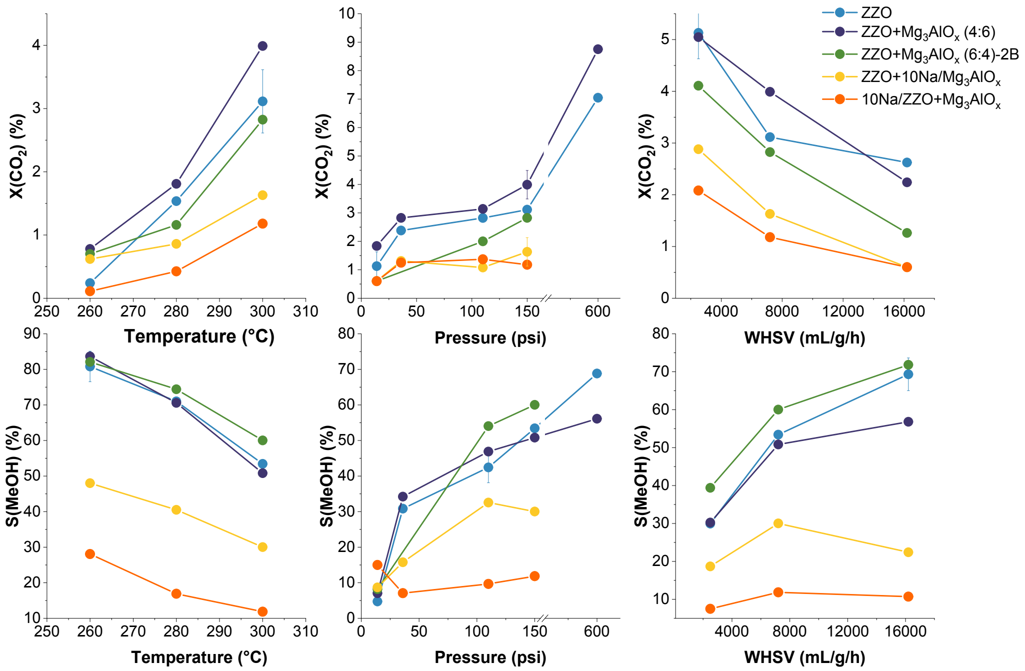

Figure S10. Steady-state co-feed CO_2 conversion and MeOH selectivity for different CS at different temperatures, pressures and WHSVs.

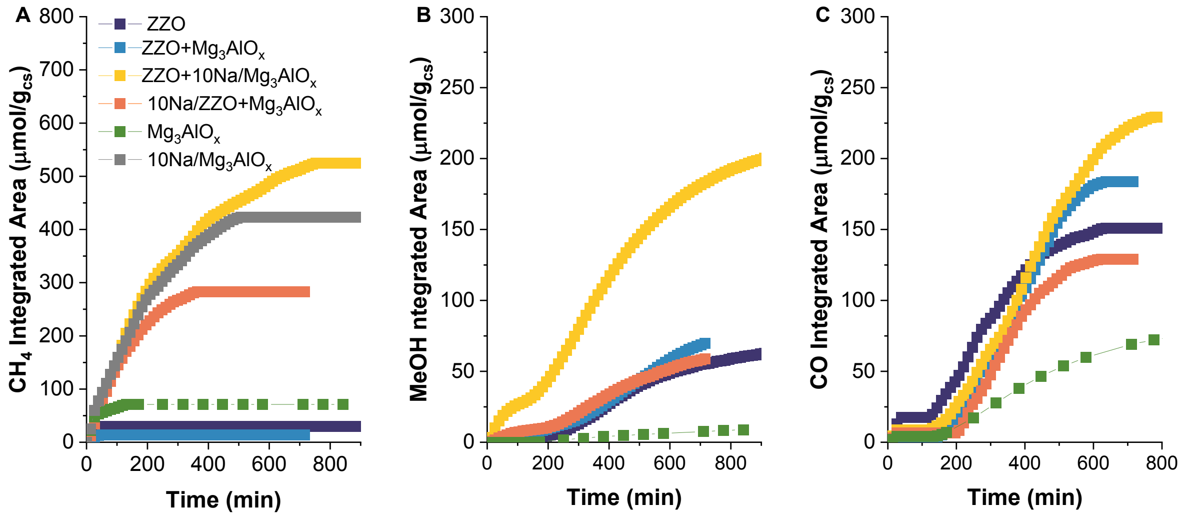

Figure S11. Rate of (A) CH_4, (B) MeOH, and (C) CO production during the conversion step of RCC at 300 °C and 6 bar for all the catalytic sorbents.

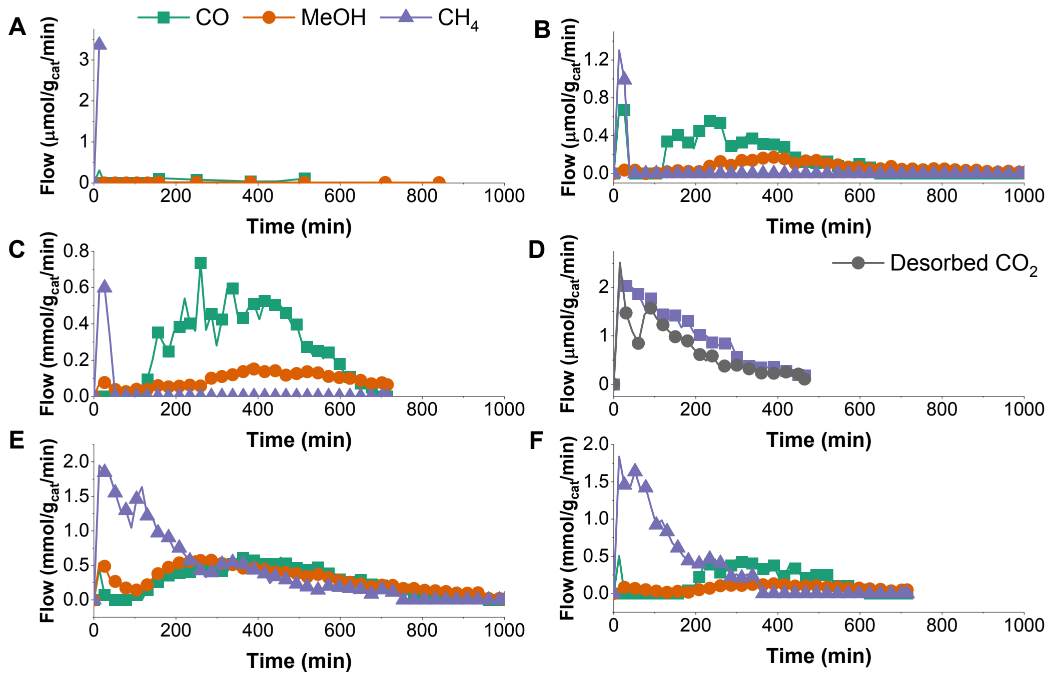

Figure S12. Flow profiles obtained during the conversion step of RCC for (A) Mg_3AlO_x, (B) ZZO, (C) ZZO+ Mg_3AlO_x, (D) 10Na/ZZO+ Mg_3AlO_x , and (D) ZZO+10Na/ Mg_3AlO_x at 300 °C and 6 bar. Each profile is the average of 2 different experiments.

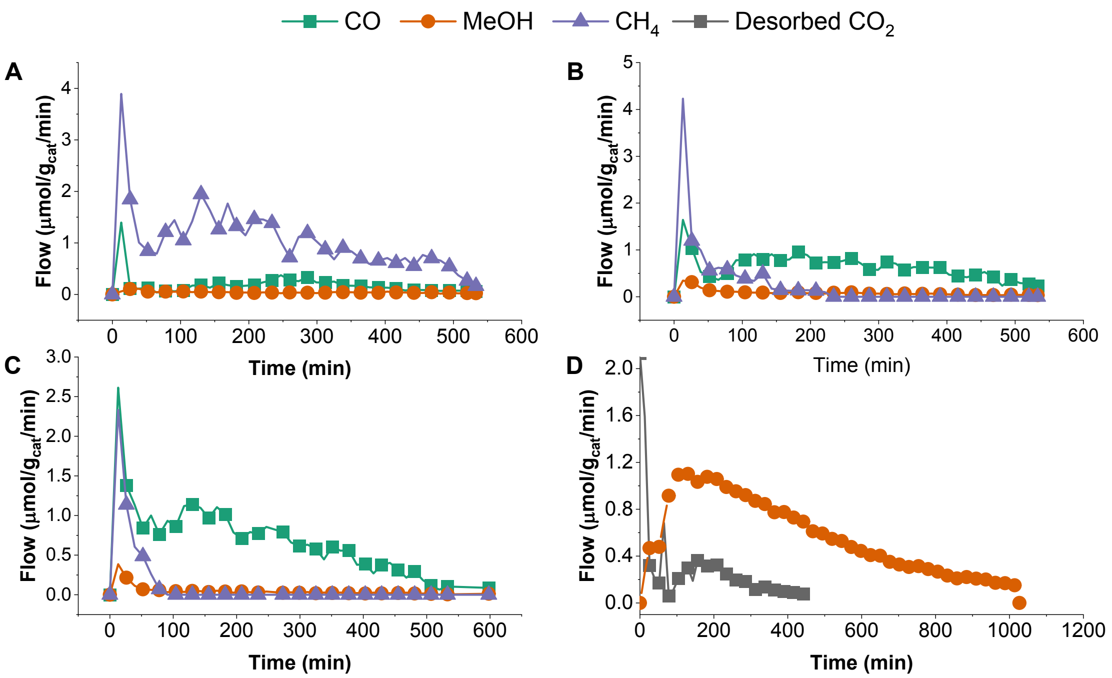

Figure S13. Flow profiles obtained during the conversion step of RCC for ZZO+10Na/ Mg_3AlO_x at 300 °C and 6 bar for (A) 2-bed configuration, (B) pelletized and mixed configuration and (C) the mixed and pelletized configuration at 320 °C and (D) the mixed and pelletized configuration at 260 °C . Each profile is the average of 2 different experiments.
